# The first Neanderthal remains from an open-air Middle Palaeolithic site in the Levant

**DOI:** 10.1038/s41598-017-03025-z

**Published:** 2017-06-07

**Authors:** Ella Been, Erella Hovers, Ravid Ekshtain, Ariel Malinski-Buller, Nuha Agha, Alon Barash, Daniella E. Bar-Yosef Mayer, Stefano Benazzi, Jean-Jacques Hublin, Lihi Levin, Noam Greenbaum, Netta Mitki, Gregorio Oxilia, Naomi Porat, Joel Roskin, Michalle Soudack, Reuven Yeshurun, Ruth Shahack-Gross, Nadav Nir, Mareike C. Stahlschmidt, Yoel Rak, Omry Barzilai

**Affiliations:** 1 Department of Physical Therapy, Faculty of Health Professions, Ono Academic College, Kiryat Ono, 55107 Israel; 2 Department of Anatomy and Anthropology, Sackler Faculty of Medicine, Tel Aviv University, Tel Aviv, 69978 Israel; 3 Institute of Archaeology, the Hebrew University of Jerusalem, Jerusalem, 91905 Israel; 4 Institute of Human Origins, Arizona State University, P.O. Box 874101, Tempe AZ, 85287-4101 Israel; 5 MONREPOS Archaeological Research Centre and Museum for Human Behavioural Evolution, Schloss Monrepos, D - 56567 Neuwied, Germany; 6 Israel Antiquities Authority, P.O. Box 586, Jerusalem, 91004 Israel; 7 Faculty of Medicine in the Galilee, Bar Ilan University, Zefat, 13115 Israel; 8 Steinhardt Museum of Natural History, Tel Aviv University, Tel Aviv, 69978 Israel; 9 Peabody Museum of Archaeology and Ethnology, Harvard University, 11 Divinity Avenue, Cambridge, MA 02138 USA; 10 Department of Cultural Heritage, University of Bologna, Via degli Ariani 1, 48121 Ravenna, Italy; 11 Department of Human Evolution, Max Planck Institute for Evolutionary Anthropology, Deutscher Platz 6, 04103 Leipzig, Germany; 12 Department of Geography & Environmental Studies, University of Haifa, Haifa, 3498838 Israel; 13 Department of Biology, University of Florence, Via del Proconsolo, 12, 50122 Firenze, Italy; 14 Luminescence Dating Lab, Geological Survey of Israel, Jerusalem, 95501 Israel; 15 Department of Maritime Civilizations, University of Haifa, Haifa, 3498838 Israel; 16 School of Sciences, Achva Academic College, Shikmim Mobile Post 79800, Shikmim, Israel; 17 Department of Diagnostic Imaging, Chaim Sheba Medical Center, Tel Hashomer, 52621 Israel; 18 Sackler Faculty of Medicine, Tel Aviv University, Tel Aviv, 69978 Israel; 19 Zinman Institute of Archaeology, University of Haifa, Haifa, 3498838 Israel; 20 School of Archaeology, University College Dublin, Belfield, Dublin 4 Ireland

## Abstract

The late Middle Palaeolithic (MP) settlement patterns in the Levant included the repeated use of caves and open landscape sites. The fossil record shows that two types of hominins occupied the region during this period—Neandertals and *Homo sapiens*. Until recently, diagnostic fossil remains were found only at cave sites. Because the two populations in this region left similar material cultural remains, it was impossible to attribute any open-air site to either species. In this study, we present newly discovered fossil remains from intact archaeological layers of the open-air site ‘Ein Qashish, in northern Israel. The hominin remains represent three individuals: EQH1, a nondiagnostic skull fragment; EQH2, an upper right third molar (RM^3^); and EQH3, lower limb bones of a young Neandertal male. EQH2 and EQH3 constitute the first diagnostic anatomical remains of Neandertals at an open-air site in the Levant. The optically stimulated luminescence ages suggest that Neandertals repeatedly visited ‘Ein Qashish between 70 and 60 ka. The discovery of Neandertals at open-air sites during the late MP reinforces the view that Neandertals were a resilient population in the Levant shortly before Upper Palaeolithic *Homo sapiens* populated the region.

## Introduction

**Figure 1:**
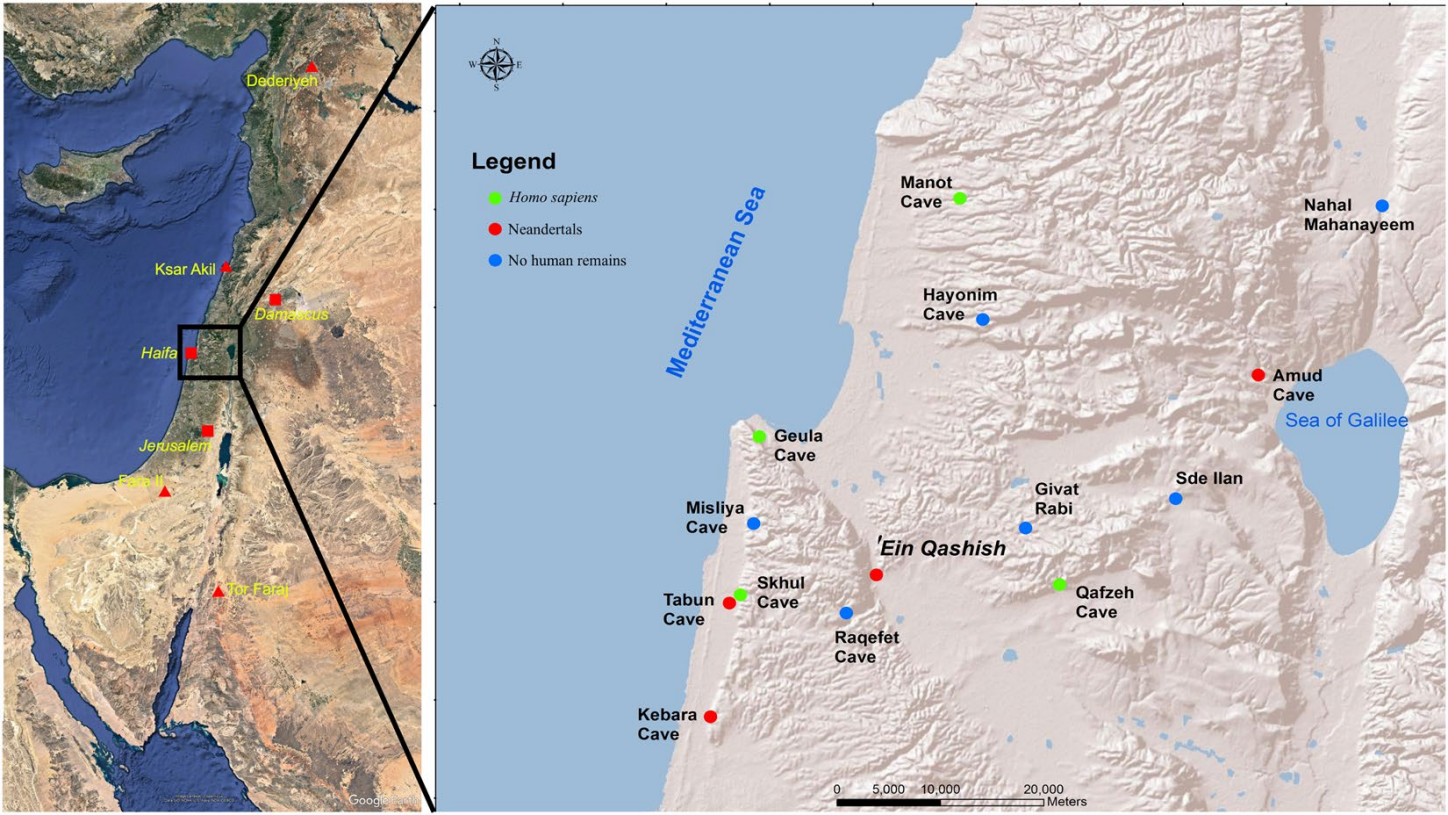
Left: Major Middle Palaeolithic sites (triangles) and modern cities (squares) in the Near East. Right: Location of ‘Ein Qashish and other Middle Palaeolithic sites in northern Israel. The map was generated using ESRI ArcInfo v10.4.

The Middle Palaeolithic (MP) of the southern Levant is a significant period for the study of human evolution because two types of hominins, Neandertals and *Homo sapiens*, occupied the region at that time (see, for example, refs 1 and 2). Diagnostic fossil remains of the two species have been found in the Mediterranean woodland region, but until recently, they were discovered only at cave sites (Fig. 1). The absolute chronology of the Levantine MP fossils indicates that *H. sapiens* existed there between 120 and 90 ka and again from 55 ka on; Neandertals existed in that region between ca. 80 and ca. 55 ka^3–16^. The genomic evidence suggests gene flow from early *H. sapiens* to the eastern Altai Neandertals ca. 100 ka^17^ and flow from Neandertals to *H. sapiens* between ca. 60 and 50 ka^18^. In the Levant, the archaeological record cannot distinguish between these two MP populations. The lithic variability observed in the Levantine MP is not clearly taxonomy related (ref. 19 for a different view see ref. 20). The two populations left similar material culture remains—in particular, lithic industries that include the Levallois technology. In addition, the populations seem to have had similar settlement and mobility patterns in respect to the use of caves for habitation and burials; at Tabun, these populations used the same cave diachronically^12, 13, 21, 22^.

The discovery of several previously unknown MP open-air sites in the Mediterranean woodland region in the last decade diverted much of the research focus to MP behaviors associated with the open landscape (e.g., ref. 23). In the absence of taxonomically informative fossil remains, it was impossible to attribute these (as well as previously reported) open-air sites to either Neandertals or *H. sapiens*. Therefore, it was also difficult to determine these species’ settlement patterns and territorial behavior within the Levant. The new discovery of Neandertal remains at the late MP open-air site of ‘Ein Qashish provides a window into the settlement and mobility patterns of the Neandertals of northern Israel.

## The Site

‘Ein Qashish is located on the south bank of the Qishon stream in the Jezreel Valley, facing the eastern slopes of Mount Carmel, Israel (Fig. 1). Excavations at the site in 2009–2011 exposed remains of a Late Mousterian occupation on the Qishon floodplain^24–28^.

**Figure 2:**
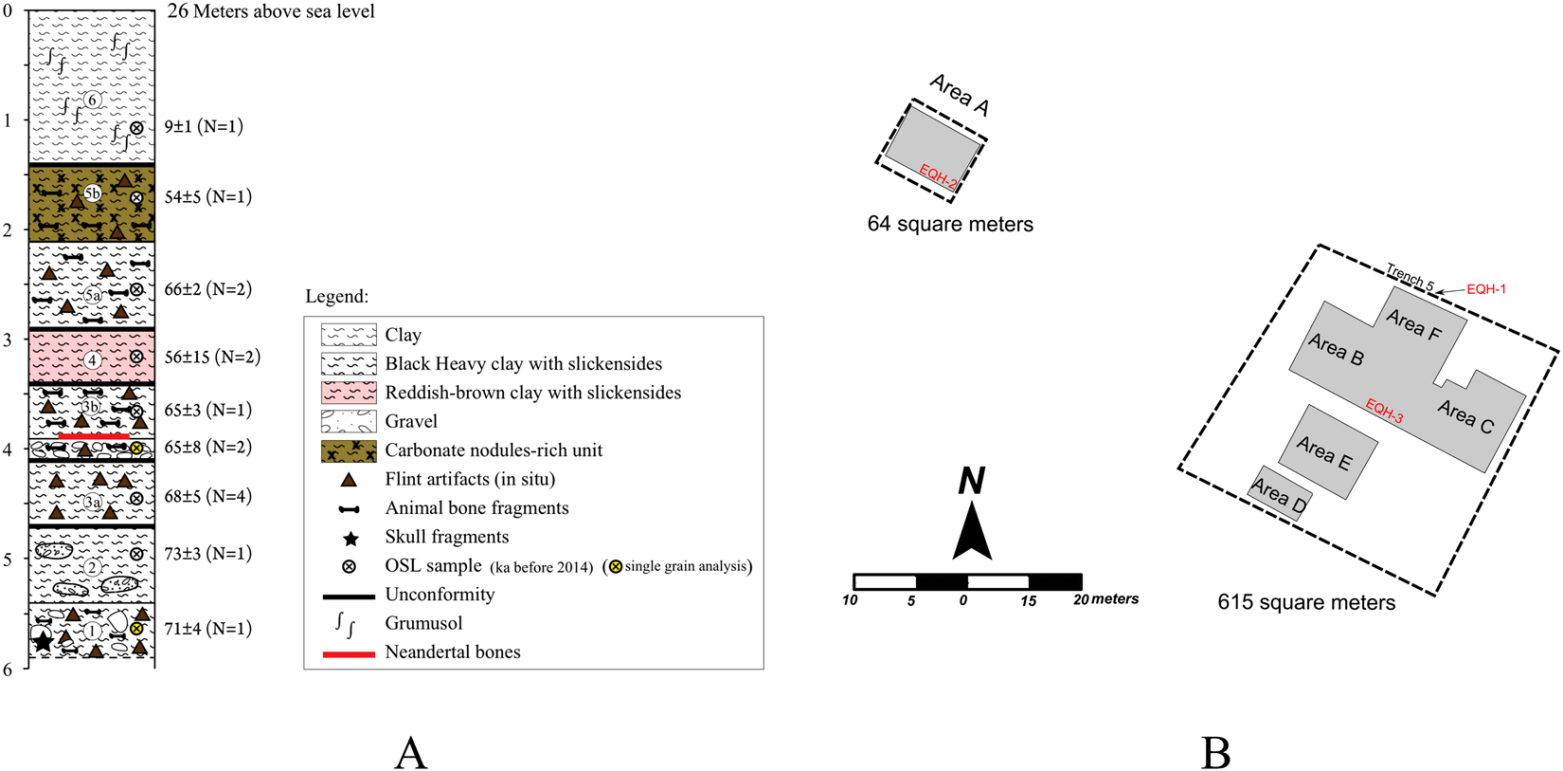
Schematic plan of ‘Ein Qashish. (**A**) Compiled stratigraphic section with vertical locations of OSL dates, in thousands of years, and hominin fossils. (**B**) Plan of excavation areas with spatial locations of hominin fossils.

In 2013, the site was subjected to an extensive salvage excavation during which an area of ca. 650 m^2^ was dug to a maximum depth of 4.5 m (ref. 29; SI 1). The stratigraphy consists of six sedimentary layers comprising four occupational horizons (Fig. 2). The 2013 excavation is laterally and stratigraphically contiguous to the original excavation, with a similar depositional context. The sediments are composed mainly of black heavy clays representing the flood plain of the palaeo-Qishon stream and coarse cobbles transported by short, steep, fast-flowing streams off the eastern flanks of Mount Carmel (refs 25 and 26; SI 1). The site sequence was dated through optically stimulated luminescence (OSL), which puts the time range of all the examined archaeological horizons at ca. 70–60 ka (SI 2 Table 1), similar to the range of dates of the stratigraphic sequence of the 2009–2011 excavation, established through the same dating techniques^26^.

### The Context of the Hominin Remains

The hominin remains from ‘Ein Qashish represent three individuals that were found in three distinct layers (Fig. 2).

Specimen EQH1 is a nondiagnostic skull fragment that was discovered in a mechanically dug geological trench prior to the 2013 excavation (ref. 29; SI 1). The stratigraphic position of the fossil corresponds to Layer 1, the lowest in the documented sections in the site’s vicinity. Layer 1 is absent from the sequence in the archaeological excavation itself (N. Greenbaum, pers. obs.). Contextual data for the layer are poor.

**Figure 3:**
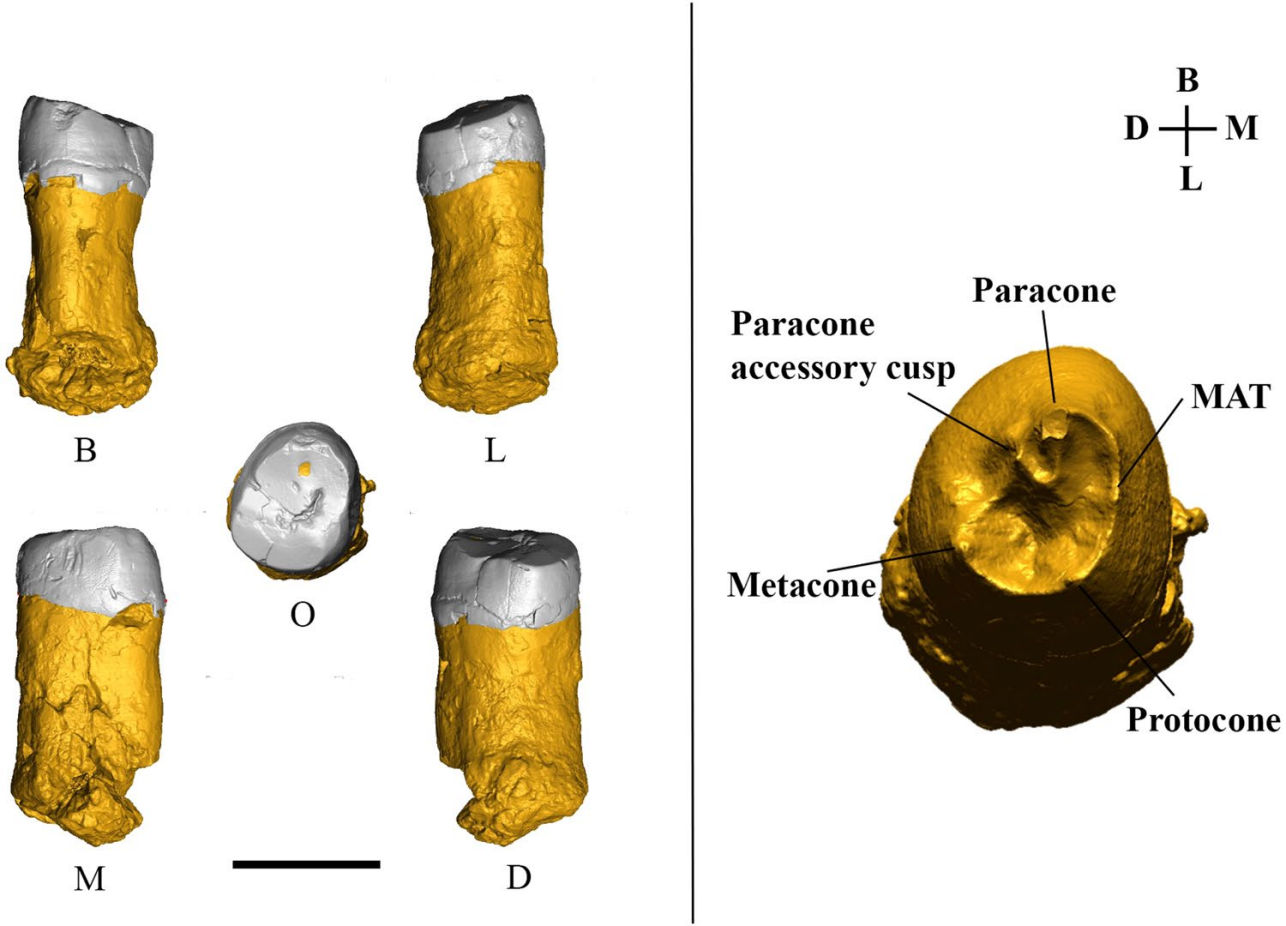
3D digital model of specimen EQH2, an upper right third molar. Left: Various views—B, buccal; L, lingual; M, mesial; D, distal; O, occlusal. The black bar represents 1 cm. Right: The enamel-dentine junction (EDJ) surface of EQH2.

The second fossil, EQH2, is an upper third molar (Fig. 3) from Layer 5a, in Area A. The fossil was found associated with flint artefacts and faunal remains in a horizon with refitted lithic items (51 refitted items in 21 aggregates) (SI 1), indicating a moderately disturbed *in situ* context.

**Figure 4:**
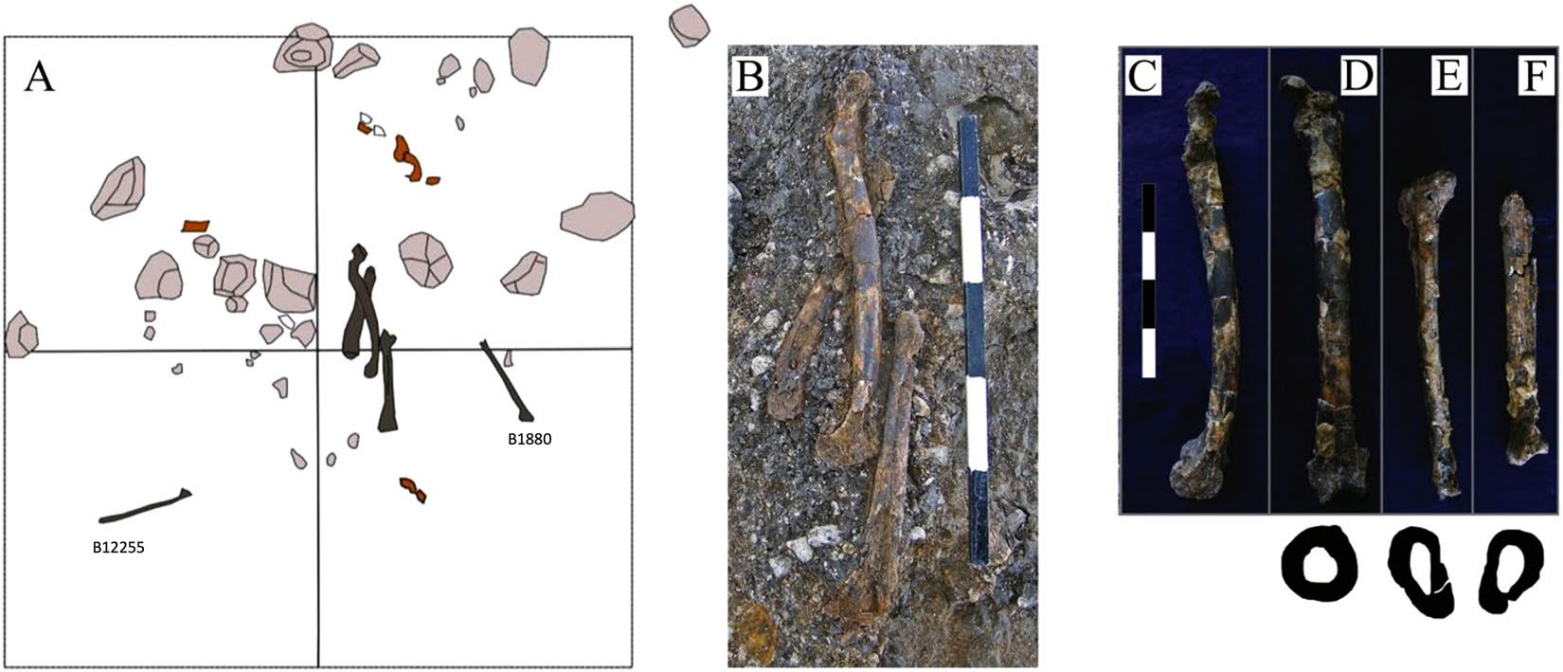
Specimen EQH3. (**A**) The spatial location of the five lower limb bones of EQH3 (dark brown); specimens B1880 and B12255 are fibulae. Pink: stones; reddish-brown: faunal remains. (**B**) The bones *in situ*. Note the partial articulation of the left femur and left tibia. (**C**) Left femur, medial view. (**D**) Left femur, anterior view; midshaft cross section (underneath). (**E**) Left tibia, anterior view; midshaft cross section (underneath). (**F**) Right tibia, anterior view; midshaft cross section (underneath).

The best-preserved specimen is EQH3, consisting of five lower limb bones—a femur, two tibiae, and two fibulae (Fig. 4)—associated with an occupational horizon in stratigraphic Layer 3b, Area B (for details of the archaeological context, see SI 1). The femur and the left tibia of EQH3 were found articulated. The bones were aligned along the same axis, with the right tibia parallel to the left (Fig. 4A,B). One of the two fibulae (B1880) was discovered ca. 50 cm north of the femur-tibia cluster, and the other fibula (B12255), ca. 70 cm south of the cluster (Fig. 4A). Finds other than human remains in this particular horizon comprise fresh flint artefacts (with 21 refitted items from four aggregates); fragmented animal bones; limestone clasts, including potential manuports (possibly anvils); ochre; a roe deer antler and a seashell, *Hexaplex trunculus* (Linnaeus, 1758) (SI 1). OSL dating of sediments directly above and below EQH3 puts the fossil at 65 ± 8 ka (SI 2).

### EQH2

EQH2 is an upper right third molar (RM^3^) on which both the crown (with a mesiodistal [MD] length of 8.3 mm and buccolingual [BL] length of 9.7 mm) and the root (with a length of 14.3 mm) are preserved. The moderate wear of the tooth, with the dentine exposed on the paracone cusp, corresponds to wear stage (category) 3 of Molnar’s dental attrition classification^30^. In an occlusal view, the crown outline is oval, and although tooth wear has removed most of the occlusal features, three main cusps (the protocone, paracone, and metacone) can be identified (Fig. 3, right). At the enamel-dentine junction (EDJ) (Fig. 3, right; SI 3 Table 1), two accessory cusps (the mesial accessory tubercle [MAT] and paracone accessory cusp) are present, but there is no trace of the distolingual cusp (the hypocone) or Carabelli’s cusp. An interproximal wear facet (length, 3.81 mm; width, 4.19 mm) is visible only on the mesial side of the tooth (Fig. 3, left). The tooth is hypertaurodontic and does not show root bifurcation.

We compared the MD and BL crown diameters of EQH2 to the diameters in tooth samples from Neandertals, early *H. sapiens*, Upper Palaeolithic *H. sapiens*, and recent *H. sapiens* (SI 3 Table 2; SI 3 Fig. 1). There is a large overlap in the distribution of the MD and BL diameters in our comparative sample. The values obtained for EQH2 are the lowest among the fossils and are closest to the values obtained for the Neandertal specimens Saccopastore 1, Amud 1, and Tabun 1 and the Upper Palaeolithic *H. sapiens* specimen Kostenki XIV (SI 3 Fig. 1). With regard to the relative enamel thickness (RET) index, the z score computed for the EQH2 RET value (18.9) is closer to the Neandertal mean than to the means of early, Upper Palaeolithic, and recent *H. sapiens* (SI 3 Tables 3, 4). Dental tissue volumes and root measurements of EQH2 and the comparative sample (SI 3 Fig. 2; SI 3 Tables 4, 5) show that the root of EQH2 is somewhat larger than in the comparative sample. The computed z score for EQH2’s root length, total root volume, pulp volume, and root pulp volume is closer to that of the Neandertals, whereas the coronal pulp volume is closer to that of Upper Palaeolithic *H. sapiens* and the cervical plane area is closer to that of recent *H. sapiens*.

The cross-validation linear discriminant analysis of four root variables (root length, root volume, pulp volume, and cervical plane area) shows that 23 modern humans (92% of our sample) and all Neandertals in our sample were correctly classified and attributes EQH2 to *H. neanderthalensis* with a *P*_post_ of 70%. Note that if we remove the cervical plane area from the analysis, EQH2 is attributed to *H. neanderthalensis* with a *P*_post_ value of 81%.

### EQH3

The lower limb bones of EQH3 consist of a left femur, two tibiae, and two fibulae. Out of the five lower limb bones, only the femur and two tibiae are preserved enough for analysis (Fig. 4C–F). The femur is essentially complete (Fig. 4). The femoral shaft is highly curved on the sagittal plane (i.e., anteroposteriorly), with the apex of the curvature located distal to the midshaft. The midshaft shape ratio (with a pilastric index of 99.1) indicates a rounded cross section (the anteroposterior diameter and mediolateral diameter are nearly equal) (Fig. 4; SI 4 Table 3). The midshaft robusticity index (14.9) indicates a highly robust femur. The midshaft cross-sectional area is large, with a relatively high percentage of cortical bone. All of these features are well-documented Neandertal characteristics that differ considerably from the more gracile femur of early and recent *H. sapiens*. There, the midshaft has a drop-shaped cross section and is straight compared to that of the Neandertals^31–36^ (SI 4). The distal epiphysis of the EQH3 femur is relatively small, and the intercondylar fossa is extremely narrow, a feature that is not usually seen in either *H. sapiens* or Neandertals (SI 4).

The remains of the right tibia include the diaphysis distal to the soleal line and the distal epiphyses; the proximal part of the tibia is missing (Fig. 4). Nearly complete, the left tibia is missing only its medial malleolus. The tibial plateau is flat, with a robust intercondylar tubercle (the medial part of the intercondylar eminence).

Both tibiae exhibit fragmented and slightly distorted shafts, which are robust and narrow mediolaterally (platycnemic), similar to the tibia of Amud 1^37^. The anterior crest of the right and left tibial shafts and the interosseous borders are smooth and rounded. At 81.5, the crural index indicates that the tibia is short relative to the femoral length. Again, most of the striking features of the tibiae are associated with Neandertal morphology: the robust shaft, the rounded anterior crest and interosseous border, and the low crural index. The morphology of these tibiae contrasts with that of the more gracile *H. sapiens* tibiae, which are characterized by generally angular anterior and interosseous crests and a high crural index^38, 39^.

The lower limb bones of EQH3 were found close together, with some in articulation. All belong to a male Neandertal, and no duplicate bones were found, suggesting that these bones represent a single individual (SI 4). The estimated height of the individual is 163.6 cm, which is close to the mean height for male Neandertals (166.7 ± 5.9 cm) and significantly less than the considerable height of the *H. sapiens* specimens from Qafzeh and Skhul (185.1 ± 7.1 cm) (SI 4; ref. 40).

Computed tomography (CT) reveals the presence of the epiphyseal line at the distal end of the femur and the proximal and distal ends of the tibiae, indicating ossification stage three out of four^41, 42^. Thus, the individual’s age at death can be estimated at 15–22 y (young adult) (SI 4).

The combination of a narrow intercondylar notch and a robust intercondylar tubercle is not often seen in the knee joints of hominins (SI 4). This unique morphology is associated with an avulsion fracture of the anterior cruciate ligament (ACL). Such a traumatic injury occurs most commonly in skeletally immature individuals, between the ages of 8 and 14 years^43^. If this pathology was present in the knee of EQH3, the individual might have suffered from instability of the left knee joint and therefore would probably have attempted to minimize the weight borne by the left leg. The small articular surface of the distal femur might be the result of the pathology, given that articular surface area is directly related to the amount of axial pressure exerted on the joint (SI 4; ref. 44).

## Discussion

The absolute dating of contexts associated with the Neandertal fossils from Tabun, Dederiyeh, Kebara, and Amud Caves suggests that Neandertals occupied the southern Levant between ca. 80 and ca. 55 ka^3, 6, 10, 12, 13, 45^. Because diagnostic hominin remains from open-air sites dated to this period were not available until now (e.g., refs 46 and 47), attributing the occupation of open-air sites to Neandertal settlement systems called for caution. However, the fossils EQH2 and EQH3 derive from two distinct stratigraphic horizons, and their associated OSL ages suggest that the open-air site of ‘Ein Qashish was used repeatedly by Neandertals from 70 to 60 ka, a period contemporary with the occupation of the Kebara and Amud Caves.

The discovery of diagnostic Neandertal remains at the open-air site of ‘Ein Qashish is unusual not only for the Levant but also for Europe, where only two sites, both of which are earlier, have yielded such diagnostic fossils: the French Tourvillel a Rivière and Biache Saint Vaast 2 sites, both dated to marine isotope stage 7^48, 49^.

The recovery of the two Neandertal fossils from ‘Ein Qashish raises questions as to the nature of their depositional histories and the inhabitants’ behavioral patterns. Whereas the tooth (EQH2) does not constitute a compelling indication of death at the site, the preservation of bones of two legs, as well as their partial articulation, suggests that the individual represented by EQH3 is likely to have died at the site or nearby. Given the bone state of preservation and articulation, the body remains must have been buried rather fast, either anthropogenically or naturally.

The presence of Neandertal fossil remains at MP sites can be interpreted as the result of intentional burial or non anthropogenic deposition (e.g., refs 50 and 56). To determine which of the scenarios applies to the ‘Ein Qashish fossils, we evaluated several parameters that may distinguish between the two scenarios: articulation, flexed position, evidence of an excavated pit, intentional coverage of the bones, and the presence of grave goods. Given the available evidence, we cannot determine whether EQH3 is a burial or not. The partial articulation of the left femur and tibia, which attests to a flexed position of the knee (Fig. 4), may support a hypothesis of intentional burial. On the other hand, there are no other body parts of the individual, no visible indication of a pit or the intentional covering of a corpse, and no grave markers. A number of uncommon finds (a seashell, roe deer antler, and ochre) that were unearthed in the same archaeological horizon are not directly associated with the bones of EQH3.

The most informative aspect of the discovery of EQH3 is that it is a Neandertal. The stratigraphic association with a diverse set of material culture remains indicates a habitation context, and the stratigraphic sequence suggests that the locality was used repeatedly. The identification of EQH2 and EQH3 enables us, for the first time, to confidently attribute to Neandertals a set of assemblages from an open-air site in the southern Levant. This discovery in the flat topography of the palaeo-Qishon flood plain demonstrates that locomotor traits did not necessarily constrain Neandertals from exploiting landscapes other than the rugged mountainous terrain (contra^57^; see also ref. 58) and, by extension, the ecological mosaic of topographically diverse environments.

Hypotheses regarding the demise of the Levantine Neandertals implicate competitive exclusion, direct competition^1, 59^, and the inability of the Neandertals to adapt to climate variability and deterioration (e.g., ref. 60). Recent studies focusing on various proxies from Kebara and Amud Caves show that climate change in the Mediterranean zone during the MIS 4 to early MIS 3 time span may not have been as drastic as suggested^61^ and that behavioral strategies enabled the Neandertals to cope with ecological change^62, 63^. Combined with the dates of the Kebara and Amud Neandertals, the repeated occupation of ‘Ein Qashish in the open landscape during the Levantine late MP reinforces the view that despite possible early interbreeding events^17^, Neandertals constituted a resilient population in the Mediterranean ecological zone of the southern Levant shortly before the region was populated by Upper Palaeolithic *H. sapiens* ^12–14, 16, 21, 64^.

## Materials and Methods

### EQH-2

High-resolution micro-CT images of EQH2 were obtained with a SkyScan1173 microtomographic system (at the Max Planck Institute for Evolutionary Anthropology, Leipzig, Germany) using the following scan parameters: 100 kV, 62 uA, with an aluminum-copper filter (1.0 mm thick). Volume data were reconstructed using isometric voxels of 12.90 µm. We segmented the image stack with a semiautomatic threshold-based approach in Avizo 8 (Visualization Sciences Group Inc.) to separate the enamel, the dentine, and the pulp chamber and to reconstruct a 3D digital model of the tooth (Fig. 3).

Before beginning the analysis, we oriented the tooth in Rapidform XOR2 software (INUS Technology, Inc., Seoul, Korea): using a spline curve, we manually digitized the cervical line and computed a best-fit plane (the cervical plane; SI 3 Fig. 2) through the points of the curve. The tooth was then rotated until the cervical plane was parallel to the *xy*-plane of the Cartesian coordinate system. The mesiodistal (MD) and buccolingual (BL) crown diameters of EQH2 were measured directly on the digital model and compared with those of Neandertals, early *H. sapiens*, Upper Palaeolithic *H. sapiens*, and recent *H. sapiens* (SI 3 Table 2; SI 3 Fig. 1).

Enamel thickness and dental tissue data were analyzed according to guidelines set by Benazzi *et al*.^65^. We measured the enamel volume (in mm^3^), dentine volume (in mm^3^, including the volume of the crown pulp chamber), and enamel-dentine junction (EDJ) surface (in mm^2^) to compute both the average enamel thickness (AET) index (the volume of enamel divided by the EDJ surface; index in millimeters) and the relative enamel thickness (RET) index (the AET index divided by the cubic root of dentine volume; a scale-free index).

For root analysis, we followed procedures provided by Kupczik and Hublin^66^. Six measurements were taken (SI 3 Fig. 2): root length (from the cervical plane to the apex of the root); total root volume (the volume of the root below the cervical plane, including dentine and pulp); pulp volume; coronal pulp volume (the portion of the pulp above the cervical plane); root pulp volume (the portion of the pulp below the cervical plane); and cervical plane area (the area of the tooth section obtained by sectioning the cervical plane).

Dental tissue data and root metrics computed for EQH2 were compared to a hominin sample that underwent micro-CT scanning at the Max Planck Institute for Evolutionary Anthropology, at a resolution ranging from 12.58 to 30.19 µm. The hominin sample consisted of M^3^ teeth from *H. heidelbergensis*, Neandertals, early *H. sapiens*, Upper Palaeolithic *H. sapiens*, and recent *H. sapiens* (SI 3 Table 3).

Standardized scores (z scores) were computed to establish which group’s mean (Neandertals, early and Upper Palaeolithic *H. sapiens*, or recent *H. sapiens*) the RET index and root metrics of EQH2 were closest to (SI 3 Table 4). Finally, we used a leave-one-out cross-validation linear discriminant analysis (LDA) of root metrics to assign the specimen to the group with the highest posterior probability.

For data processing and analyses, we used R software v. 2.15.1^67^.

### EQH3

Femoral and tibial length dimensions were obtained with a sliding caliper and osteometric board. For angular measurements, we used a goniometer (SI 4 Tables 1, 2; SI 4 Figs 1,2). Osteological measurements follow those defined by Martin^68^ and other scholars^35, 36, 69^. The bones were scanned on a medical CT scanner at standard medical calibration (120 kV; 0.5 mm thick layers) at the Sheba Medical Center in Israel. The total cross-sectional area and total cross-sectional area of the cortical bone were measured at the reformatted horizontal plane of the femoral midshaft. The illustrations of the midshaft cross sections that appear in Fig. 4D,E,F (below the photographs) are based on the horizontal reformatted cross sections of the three bones. A specialist in pediatric radiology (MS) identified the epiphyseal line on the CT scans and noted the presence and absence of pathologies.

The results for EQH3 were compared to those for recent *H. sapiens*, early *H. sapiens*, and *H. neanderthalensis*, taken from published data (for example, refs 35 and 36; see SI 4 Tables 3,4). Well-established morphological differences between the femur and tibia of Neandertals and *H. sapiens* enabled us to identify EQH3 as a Neandertal (SI 4). Age estimation was based on the stage of epiphyseal union, bone length, and age-related pathology (osteoarthritis) (SI 4). We determined gender on the basis of morphological differences between male and female Neandertals (SI 4 Table 5)^33, 69^. The stature estimation was based on 11 formulas: three formulas use femur length, four use tibial length, and four use femoral and tibial length (SI 4 Table 6). EQH3’s stature was compared to that of recent *H. sapiens*, early *H. sapiens*, *H. neanderthalensis*, and the Sima de los Huesos hominins (SI 4 Table 7).

## Electronic supplementary material

Supplementary information

## References

[1] Shea JJ (2003). The Middle Paleolithic of the East Mediterranean Levant. J. World Prehist..

[2] Hovers, E. Neandertals and modern humans in the Middle Paleolithic of the Levant: What kind of interaction? *When Neandertals and Moderns Met*, (ed. Conard, N.) 65–86 (Kerns Verlag, Tübingen, 2006).

[3] Valladas H (1987). Thermoluminescence dates for the Neanderthal burial site at Kebara in Israel. Nature.

[4] Valladas H (1988). Thermoluminescence dating of Mousterian ‘Proto-Cro-Magnon’ remains from Israel and the origin of Modern Man. Nature.

[5] Valladas, H., Mercier, N., Joron, J. L. & Reyss, J. L. GIF Laboratory dates for Middle Paleolithic Levant. *Neandertals and Modern Humans in Western Asia*, (eds Akazawa, T., Aoki, K., Bar-Yosef, O.) 69–76 (Plenum Press, New York, 1998).

[6] Valladas H (1999). TL Dates for the Neandertal site of Amud Cave, Israel. J. Archaeol. Sci..

[7] Bar-Yosef O, Callander J (1999). The woman from Tabun: Garrod’s doubts in historical perspective. J. Hum. Evol..

[8] Grün R, Stringer CB (2000). Tabun Revisited: Revised ESR Chronology and New ESR and U-series Analyses of Dental Material from Tabun C1. J. Hum. Evol..

[9] Stringer CB, Grün R, Schwarcz HP, Goldberg P (1989). ESR dates for the hominid burial site of Es Skhul in Israel. Nature.

[10] Mercier N (1993). Thermoluminescence date for the Mousterian burial site of Es-Skhul, Mt. Carmel. J. Archaeol. Sci..

[11] Mercier N, Valladas H (2003). Reassessment of TL age estimates of burnt flints from the Paleolithic site of Tabun Cave, Israel. J. Hum. Evol..

[12] Coppa A, Grün R, Stringer C, Eggins S, Vargiu R (2005). Newly recognized Pleistocene human teeth from Tabun Cave, Israel. J. Hum. Evol..

[13] Coppa A, Manni F, Stringer C, Vargiu R, Vecchi F (2007). Evidence for new Neanderthal teeth in Tabun Cave (Israel) by the application of self-organizing maps (SOMs). J. Hum. Evol..

[14] Bosch MD (2015). New chronology for Ksâr ‘Akil (Lebanon) supports Levantine route of modern human dispersal into Europe. Proc. Natl. Acad. Sci. USA.

[15] Hovers, E. *The Lithic Assemblages of Qafzeh Cave*. (Oxford University Press, Oxford, 2009).

[16] Hershkovitz I (2015). Levantine cranium from Manot Cave (Israel) foreshadows the first European modern humans. Nature.

[17] Kuhlwilm M (2016). Ancient gene flow from early modern humans into Eastern Neanderthals. Nature.

[18] Fu Q (2014). Genome sequence of a 45,000-year-old modern human from western Siberia. Nature.

[19] Hovers E, Belfer-Cohen A (2013). On variability and complexity: lessons from the Levantine Middle Paleolithic record. Curr. Anthropol..

[20] Groucutt HS (2015). Stone tool assemblages and models for the dispersal of Homo sapiens out of Africa. Quat. Int..

[21] McCown, T. D. & Keith, A. *The Stone Age of Mount Carmel II*. (Clarendon Press, Oxford, 1939).

[22] Rak, Y. Does any Mousterian cave present evidence of two hominid species? *Neandertals and Modern Humans in Western Asia*, (eds Akazawa, T., Aoki, K., Bar-Yosef, O.) 353–366 (Plenum Press, New York, 1998).

[23] Sharon G, Zaidner Y, Hovers E (2014). Opportunities, problems and future directions in the study of open air Middle Paleolithic sites. Quat. Intern..

[24] Hovers E, Malinsky-Buller A, Ekshtain R, Oron M, Yeshurun R (2008). ‘Ein Qashish—a new open air Middle Paleolithic site in northern Israel. J. Israel. Prehist. Soc..

[25] Hovers E (2014). Islands in a stream? Reconstructing site formation processes in the late Middle Paleolithic site of ‘Ein Qashish, northern Israel. Quat. Intern..

[26] Greenbaum N, Ekshtain R, Malinsky-Buller A, Porat N, Hovers E (2014). The stratigraphy and paleogeography of the Middle Paleolithic open air site of ‘Ein Qashish, Northern Israel. Quat. Intern..

[27] Ekshtain R, Malinsky-Buller A, Ilani S, Segal I, Hovers E (2014). Raw material exploitation around the Middle Paleolithic site of ‘Ein Qashish. Quat. Intern..

[28] Malinsky-Buller A, Ekshtain R, Hovers E (2014). Organization of lithic technology at ‘Ein Qashish, a late Middle Paleolithic open air site in Israel. Quat. Intern..

[29] Barzilai, O., Ekshtain, R., Malinsky-Buller, A. & Hovers, E. ‘En Qashish (‘Ein Qashish). *Hadashot Arkheologiot* **127** (2015) http://www.hadashot-esi.org.il.

[30] Molnar S (1971). Human tooth wear, tooth function and cultural variability. Am. J. Phys. Anthropol..

[31] Boule, M. L’homme fossile de La Chapelle-aux-Saints (Vol. 6) (Masson,1913).

[32] Trinkaus, E. The evolution of the hominid femoral diaphysis during the Upper Pleistocene in Europe and the Near East. *Zeitschrift für Morphologie und Anthropologie* 291–319 (1976). 827134

[33] Trinkaus, E. *The Shanidar Neandertals* (Academic Press, New York, 1983).

[34] Heim, J. L. Les Hommes Fossiles de La Ferrassie. Tome II. Les Squelettes adultes (squelette de membres). *Arch. Inst. Paléontol. Humaine*. **38** (1982).

[35] Shackelford LL, Trinkaus E (2002). Late Pleistocene human femoral diaphyseal curvature. Am. J. Phys. Anthropol..

[36] De Groote I (2011). Femoral curvature in Neanderthals and modern humans: a 3D geometric morphometric analysis. J. Hum. Evol..

[37] Lovejoy CO, Trinkaus E (1980). Strength and robusticity of the Neandertal tibia. Am. J. Phys. Anthropol..

[38] Stringer CB, Trinkaus E, Roberts MB, Parfitt SA, Macphail RI (1998). The middle Pleistocene human tibia from Boxgrove. J. Hum. Evol..

[39] Trinkaus E, Ruff CB (1999). Diaphyseal cross-sectional geometry of Near Eastern Middle Palaeolithic humans: the femur. J. Archaeol. Sci..

[40] Carretero JM (2012). Stature estimation from complete long bones in the Middle Pleistocene humans from the Sima de los Huesos, Sierra de Atapuerca (Spain). J. Hum. Evol..

[41] O’Connor JE, Bogue C, Spence LD, Last J (2008). A method to establish the relationship between chronological age and stage of union from radiographic assessment of epiphyseal fusion at the knee: an Irish population study. J. Anatom..

[42] Dogaroiu C, Avramoiu M (2015). Correlation between chronological age and the stage of union of the distal femur and proximal tibia epiphyses in a Romanian sample population. Rom. J. Leg. Med..

[43] Zionts, L.E. Fractures and dislocations about the knee. *Skeletal Trauma in Children 3rd ed*. 439–471 (Philadelphia, PA: Saunders, 2003).

[44] Swartz SM (1989). The functional morphology of weight bearing: limb joint surface area allometry in anthropoid primates. J. Zool.

[45] Kadowaki, S. Issues of Chronological and Geographical Distributions of Middle and Upper Palaeolithic Cultural Variability in the Levant and Implications for the Learning Behavior of Neanderthals and Homo sapiens. *Dynamics of Learning in Neanderthals and Modern Humans Volume 1: Cultural**Perspectives, Replacement of Neanderthals by* Modern Humans Series (eds Akazawa, T. *et al*.) 59–91, doi:10.1007/978-4-431-54511-8_4 (Springer Japan, 2013).

[46] Sharon G, Oron M (2014). The lithic tool arsenal of a Mousterian hunter, *Quarter*. Intern..

[47] Ziaei, M., Schwarcz, H. P., Hall, C. M. & Grün, R. Radiometric dating of the Mousterian site at Quneitra. *Quneitra: A Mousterian Site on the Golan Heights*. 232–235 (The Hebrew University, Jerusalem, 1990).

[48] Guipert G, de Lumley MA, Tuffreau A, Mafart B (2010). A late Middle Pleistocene hominid: Biache-Saint-Vaast 2, north France. CR Palevol..

[49] Faivre JP (2014). Middle Pleistocene Human Remains from Tourville la Rivière (Normandy, France) and Their Archaeological Context. PLoS ONE.

[50] Belfer-Cohen A, Hovers E (1992). In the eye of the beholder: Mousterian and Natufian burials in the Levant. Curr. Anthropol..

[51] Gargett RH (1989). Grave shortcomings: the evidence for neandertal burial. Curr. Anthropol..

[52] Gargett R (1999). Middle Palaeolithic burial is not a dead issue: The view from Qafzeh, Saint-Césaire, Kebara, Amud, and Dederiyeh. J. Hum. Evol..

[53] Hovers E, Kimbel WH, Rak Y (2000). Amud 7—still a burial. Response to Gargett. J. Hum. Evol..

[54] Zilhão, J. Lower and Middle Palaeolithic mortuary behaviours and the origins of ritual burial. *Death Rituals, Social Order and the Archaeology of Immortality in the Ancient World. ‘Death Shall Have No Dominion’* (eds Renfrew, C., Boyd, M. J., Morley, I.) 27–44 (Cambridge University Press, 2015).

[55] Rendu W (2014). Evidence supporting an intentional Neandertal burial at La Chapelle-aux-Saints. Proc. Natl. Acad. Sci. USA.

[56] Dibble HL (2015). A critical look at evidence from La Chapelle-aux-Saints supporting an intentional Neandertal burial. J. Archaeol. Sci..

[57] Henry DO (2016). The effect of terrain on Neanderthal ecology in the Levant. Quat. Int..

[58] Higgins RW, Ruff CB (2011). The effects of distal limb segment shortening on locomotor efficiency in sloped terrain: implications for Neandertal locomotor behavior. Am. J. Phys. Anthropol..

[59] Rak, Y. Morphological variation in Homo neanderthalensis and Homo sapiens in the Levant: a biogeographical model. *Species, Species Concept and Primate Evolution* (eds Kimbel, W. H. and Martin, L. B.) 523–536 (Plenum Press, New York, 1993).

[60] Shea JJ (2008). Transitions or turnovers? Climatically-forced extinctions of Homo sapiens and Neanderthals in the east Mediterranean Levant. Quat. Sci. Rev..

[61] Belmaker M, Hovers E (2011). Ecological change and the extinction of the Levantine Neanderthals: implications from a diachronic study of micromammals from Amud Cave, Israel. Quat. Sci. Rev..

[62] Speth, J. D. & Clark, J. Hunting and overhunting in the Levantine Late Middle Palaeolithic. *Before Farming* **3**, Article 1 (2006).

[63] Hartman G (2015). Isotopic evidence for Last Glacial climatic impacts on Neanderthal gazelle hunting territories at Amud Cave, Israel. J. Hum. Evol..

[64] Hublin JJ (2015). The modern human colonization of western Eurasia: when and where?. Quat. Sci. Rev..

[65] Benazzi S (2014). Technical Note: Guidelines for the Digital Computation of 2D and 3D Enamel Thickness in Hominoid Teeth. Am. J. Phys. Anthropol..

[66] Kupczik K, Hublin JJ (2010). Mandibular molar root morphology in Neanderthals and Late Pleistocene and recent Homo sapiens. J. Hum. Evol..

[67] R Development Core Team. R: a language and environment for statistical computing. R Foundation for Statistical Computing, Vienna, Austria. http://www.r-project.org. (2012).

[68] Martin, R. *Lehrbuch der Anthropologie in Systematischer Darstellung mit Besonderer Berücksichtigung der Anthropologischen Methoden für Studierende, Ärtze und Forschungsreisende* (Zweiter Band: Kraniologie, Osteologie, 2nd ed. Jena, Gustav Fischer, 1928).

[69] Trinkaus E (1980). Sexual differences in Neanderthal limb bones. J. Hum. Evol..

