# Supplementary material for: The first Neanderthal remains from an open-air Middle Palaeolithic site in the Levant
Source: Sci Rep. 2017 Jun 7;7:2958. doi: 10.1038/s41598-017-03025-z (PMC5462778; doi:10.1038/s41598-017-03025-z)
Supplement: Supplementary file 1 — Supplementary information [file 41598_2017_3025_MOESM1_ESM.pdf]

The first Neanderthal remains from an open-air Middle Palaeolithic site in the Levant

Supporting Information

SI 1.'Ein Qashish: Site Background

SI 2. Optically Stimulated Luminescence (OSL) Dating

SI 3. EQH-2 (Upper Third Molar)

SI 4. Description of the EQH-3 Bones

Ella Been[1✉,2], Erella Hovers[3,4], Ravid Ekshtain[3], Ariel Malinski-Buller[5], Nuha Agha[6], Alon Barash[7], Daniella E. Bar-Yosef Mayer[8,9], Stefano Benazzi[10,11], Jean-Jacques Hublin[11], Lihi Levin[2], Noam Greenbaum[12], Netta Mitki[3], Gregorio Oxilia[13,10], Naomi Porat[14], Joel Roskin[15,16], Michalle Soudack[17,18], Reuven Yeshurun[19], Ruth Shahack-Gross[15], Nadav Nir[3], Mareike C. Stahlschmidt[20], Yoel Rak[2], Omry Barzilai[6]

[1] Department of Physical Therapy, Faculty of Health Professions, Ono Academic College, Kiryat Ono 55107, Israel

[2] Department of Anatomy and Anthropology, Sackler Faculty of Medicine, Tel Aviv University, Tel Aviv 69978, Israel

[3] Institute of Archaeology, the Hebrew University of Jerusalem, Jerusalem 91905, Israel

[4] Institute of Human Origins, P.O. Box 874101, Arizona State University, Tempe AZ 85287-4101 USA

[5] MONREPOS Archaeological Research Centre and Museum for Human Behavioural Evolution, Schloss Monrepos D – 56567 Neuwied, Germany

[6] Israel Antiquities Authority, P.O. Box 586, Jerusalem 91004, Israel

[7] Faculty of Medicine in the Galilee, Bar Ilan University, Zefat 13115, Israel

[8] Steinhardt Museum of Natural History, Tel Aviv University, Tel Aviv 69978, Israel

[9] Peabody Museum of Archaeology and Ethnology, Harvard University, 11 Divinity Avenue Cambridge MA 02138, USA

[10] Department of Cultural Heritage, University of Bologna, Via degli Ariani 1 48121 Ravenna, Italy

[11] Department of Human Evolution, Max Planck Institute for Evolutionary Anthropology, Deutscher Platz 6 04103 Leipzig, Germany

[12] Department of Geography & Environmental Studies, University of Haifa, Haifa 3498838, Israel

[13] Department of Biology, University of Florence, Via del Proconsolo, 12 50122 Firenze, Italy

[14] Luminescence Dating Lab, Geological Survey of Israel, Jerusalem 95501, Israel

[15] Department of Maritime Civilizations, University of Haifa, Haifa 3498838, Israel

[16] School of Sciences, Achva Academic College, Shikmim Mobile Post 79800, Israel

[17] Department of Diagnostic Imaging, Chaim Sheba Medical Center, Tel Hashomer 52621, Israel

[18] Sackler Faculty of Medicine, Tel Aviv University, Tel Aviv 69978, Israel

[19] Zinman Institute of Archaeology, University of Haifa, Haifa 3498838, Israel

[20] School of Archaeology, University College Dublin, Belfield Dublin 4, Ireland

SI 1. ‘Ein Qashish: Site Background

The site complex of ‘Ein Qashish is located in the Jezreel Valley, ca. 100 m south of the Qishon stream and south of Tel Qashish (SI 1 Fig.1). The site was discovered in a 2004 survey by the Israel Antiquities Authority (IAA). It was excavated during 2005 (24), and again during 2009, 2010 and 2011, by the Hebrew University of Jerusalem (HUJ) (25). The excavations revealed remains of a late Mousterian campsite dated to 70,000–60,000 years ago (26–28). A nearly complete horned skull of an auroch (*Bos primigenius*) was found associated with flint tools during this excavation. Deposition of artefacts and bones was partially secondary, and the original position of the site was hypothesized, on the basis of lithic taphonomy and geomorphological considerations, to be 40–50 m south-southwest of the excavation area (25).

In preparation for the extension of a major highway in the region, archaeological prospection was carried out by the IAA in the site’s vicinity to determine its potential and decide on excavation plans. Mechanical test trenches were dug in 2012 to estimate the size of the site (29). The trenches revealed that the Middle Palaeolithic site extended over an area of more than 1400 m^2 (SI 1 Fig. 1). Notably, a nondiagnostic fragment of a human skull was recovered from the base of Trench 5 in waterlogged sediments corresponding to geological Layer 1 (SI 1 Fig. 1).

Accidental damage to the site led to a large-scale salvage excavation in the summer of 2013. The excavation, a collaboration between the HUJ and the IAA, was conducted in six Areas (A–F), totaling ~650 m^2 (SI 1 Fig. 2). All the areas were excavated according to a single grid system and aligned to the Israel Grid System coordinates.

All artefacts larger than 20 mm were measured three-dimensionally using Total Station instruments (Sokkia 630 and FTD 05). Other artefacts were collected and bagged according to 50 × 50 cm subsquares and 5 cm spits. All the sediments were dry-sieved and 10–15% were wet-sieved.

Stratigraphy and Sedimentology

The sedimentary sequence exposed in the 2013 excavation was tentatively correlated to a sedimentary sequence documented in geological trenches dug in 2012 (Greenbaum, pers. obs.), which included six layers (Fig. 2). The two lowermost layers were not exposed in the current excavation; thus, the site's sequence begins with Layer 3.

In general, sediments in all layers are dominated by clay but contain also silt and fine sand, rich in quartz. Layer 3 is very rich in stone and bone artefacts, including the remains of EQH-3 in Layer 3b. The layer is composed of dark black, clay-rich sediment (Layer 3a) that grades vertically into gray-black, clay-rich sediment (Layer 3b). The sedimentary layer has abundant slickensides and metallic gley along cracks. This field appearance indicates hydromorphic reducing conditions. In addition, this layer includes fossilized roots and rootlets (rhizoliths), gypsum crystals, occasional calcite veins along cracks, and cobbles from local geological formations. These indicate that the sediments were previously exposed long enough to allow for plant growth (rhizoliths) and evaporation (gypsum), i.e., a habitat conducive to human activity.

Infrared, x-ray fluorescence (XRF), and inductively coupled plasma (ICP) mass spectrometry analyses of minerals and elements in animal bones from this unit

show an abundance of iron and manganese oxides. These often appear as dark or reddish-orange coats on and within the bones. Bone mineral crystallinity was evaluated using the infrared splitting factor method. Values measured in 84 samples of animal bones average 3.4+/_0.2, indicating moderately well preserved bone mineral. The human femur bone mineral crystallinity falls within the same range. All the bones are devoid of collagen (70).

Layer 3 grades into the overlying Layer 4, a reddish brown, clay-rich unit up to ca. 50 cm thick that contains rhizoliths and gypsum crystals. The spatial extent of this unit is more confined than that of the underlying and overlying layers, and Middle Palaeolithic (MP) artefacts are fewer.

The Archaeological Context of the Hominin Finds

Area A, where specimen EQH-2 was found, is situated ca. 30 m west of the area of the excavations carried out during the years 2009, 2010, and 2011, and it may represent the original locality from where the finds were fluvially transported (25). Forty-two m^2 were excavated in this area, exposing two Middle Palaeolithic layers: Layer 5a, 60–90 cm thick, and Layer 5b, 50–70 cm thick. Both layers were cut at the northeastern corner of the area by a Holocene fluvial channel (29). The finds from Layer 5b are abundant and consist of fresh, sharp flint items in a variety of sizes, as well as fragments of animal bones and teeth belonging to large herbivores, mainly auroch. The flint assemblage from this layer is characterized by large primary items showing the initial stages of the knapping sequences. Twenty-one aggregates containing 51 artefacts have been identified so far (work in progress), showing several reduction sequences (SI 1 Fig. 3). Refits from one aggregate were found at maximum

vertical distances of 50 cm and maximum horizontal distances of 4 m, suggesting limited postdepositional vertical dispersion. EQH-2 was found in Layer 5a, which yielded fewer finds than its overlying layer, 5b. Lithic production technology was the same as in Layer 5b.

Area B is situated at the northwestern part of the excavation plot. Ca. 45 m^2 were excavated, exposing a well-preserved horizon ca. 20–40 cm thick (Layer 3b). Specimen EQH-3 was found in this layer (SI 1 Fig. 4), which also contained many flint items (mostly in fresh condition with only a few abraded or patinated), fragmented animal bones, ochre, a complete antler of a roe deer, and a marine mollusk from the Mediterranean Sea, *Hexaplex trunculus* (Linnaeus, 1758) (SI 1 Fig. 5a). Among the large angular cobbles found embedded in the clay were three modified stones that might have functioned as anvils (detailed analysis and residue analysis are in progress) (SI 1 Fig. 5b). These items were found at 22.25–22.20 m above mean sea level, at the same elevation as the EQH-3 bones but several meters from them.

Preliminary refitting efforts in Area B have so far resulted in 4 aggregates composed of 21 flint pieces. Of these, 8 aggregates derive from an artefact concentration in squares L44–L45, at an elevation of 21.96–22.08 m (ca. 3 m north of the remains of EQH-3), with little horizontal (up to 1 m) or vertical (up to 12 cm) movement. The additional aggregate of 2 pieces in squares I41–J41, 21.83–21.86 also shows little horizontal (1 m) or vertical movement (3 cm).

Lithics

All the lithic artefacts from the 2013 excavation were assigned to the Middle Palaeolithic. The densities of lithic artefacts in the various excavation areas differ and may correspond to differences in depositional and postdepositional conditions. The technological makeup of the lithic assemblages is similar, including the Levallois component (SI 1 Fig. 6). The frequencies of Levallois flaking are similarly low in all the excavated areas, a pattern known in many Levantine open-air sites (15 and references therein).

Fauna

A preliminary count and analysis of specimens identifiable to the genus or species level (n = 87) yielded a medium-sized faunal assemblage completely dominated by ungulates; additionally, more than 200 faunal specimens were identified to the level of anatomical part and body-size class. No small game or carnivore skeletal elements were found. The most frequent species is the auroch (*Bos primigenius*), followed by the mountain gazelle (*Gazella gazella*), Mesopotamian fallow deer (*Dama mesopotamica*), equid (*Equus* sp.), wild boar (*Sus scrofa*), and roe deer (*Capreolus capreolus*). The excavations of 2009, 2010, and 2011 yielded a similar faunal spectrum, dominated by auroch, Mesopotamian fallow deer, and mountain gazelle (25).

Differences were noted in the density of the faunal remains and in their degree of breakage and abrasion within the excavation areas. Most notable were the differences in the density, breakage, and abrasion of remains in Area C, which yielded only a handful of small, weathered fragments, and Areas B and F, which included hundreds of well-preserved, identifiable bones. Some between-area differences were

noted in the taxonomic spectrum, specifically the dominance of auroch in Area A and the more even representation of the three major ungulate species in Area B.

Skeletal parts rich in meat, such as upper limb bones, were found in all the excavation areas. Virtually all the limb bones are fragmented, and some show cut marks, typical of meat filleting, as well as hammerstone percussion marks (SI 1 Fig. 7). This indicates that human agents were responsible for the deposition of the animal remains, most probably the result of hunting.

SI 1 Figures

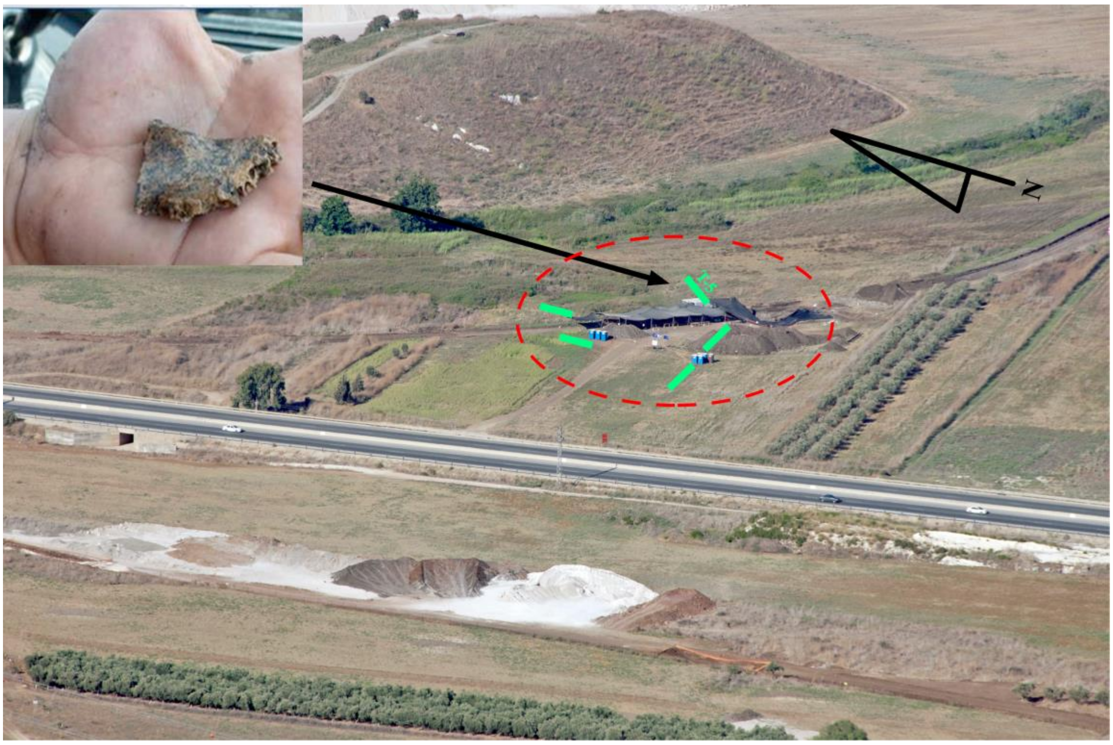

SI 1 Fig. 1. The 2013 excavation at 'Ein Qashish (area under the shade cloth). Locations of the 2009–2011 excavations, and of the 2012 mechanical trenches (in green). The estimated area of the site (dotted red circle) and the location of EQH-1 are indicated.

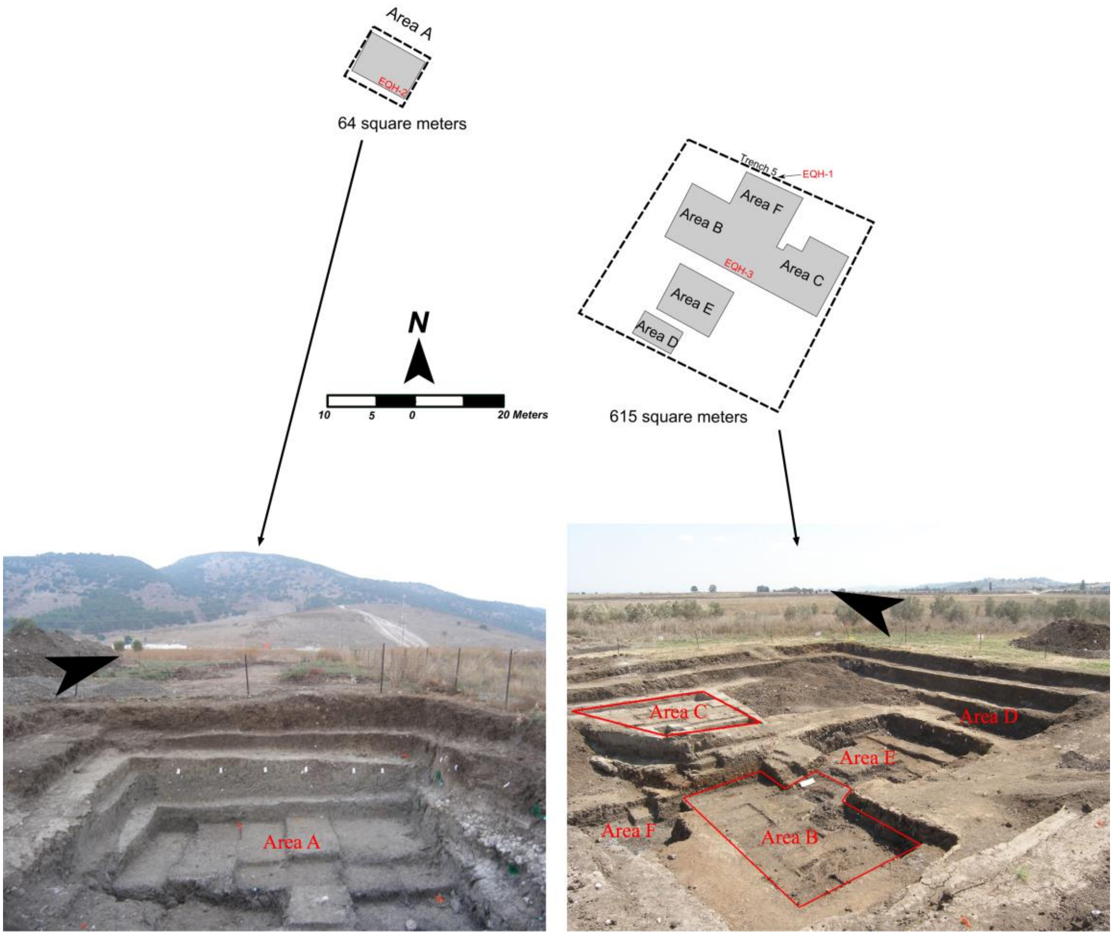

SI 1 Fig. 2. The excavation areas in ‘Ein Qashish (2013).

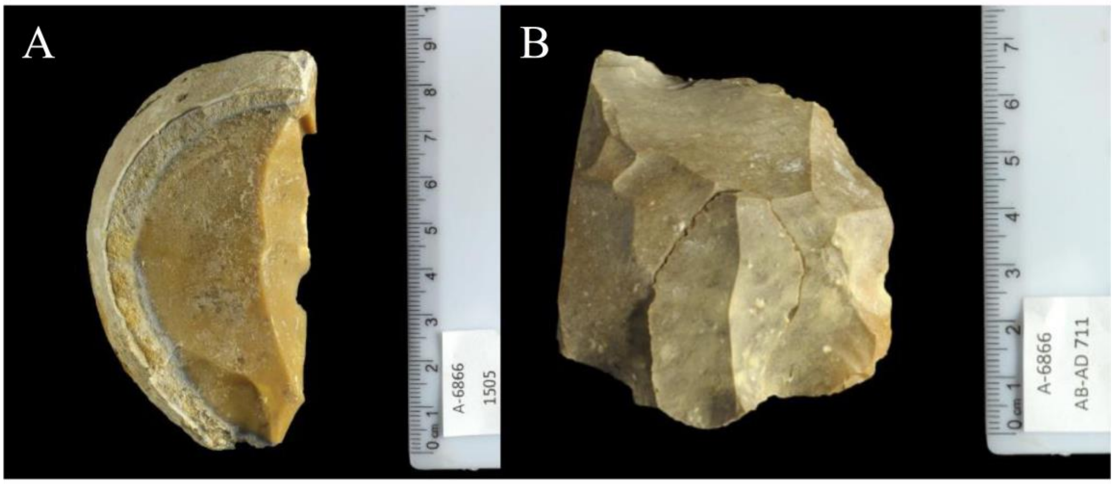

SI 1 Fig. 3. Flint artefacts from Area A. (A) A large flake with cortex. (B) A refitted flint aggregate.

Photo Clara Amit, Courtesy of the Israel Antiquities Authority

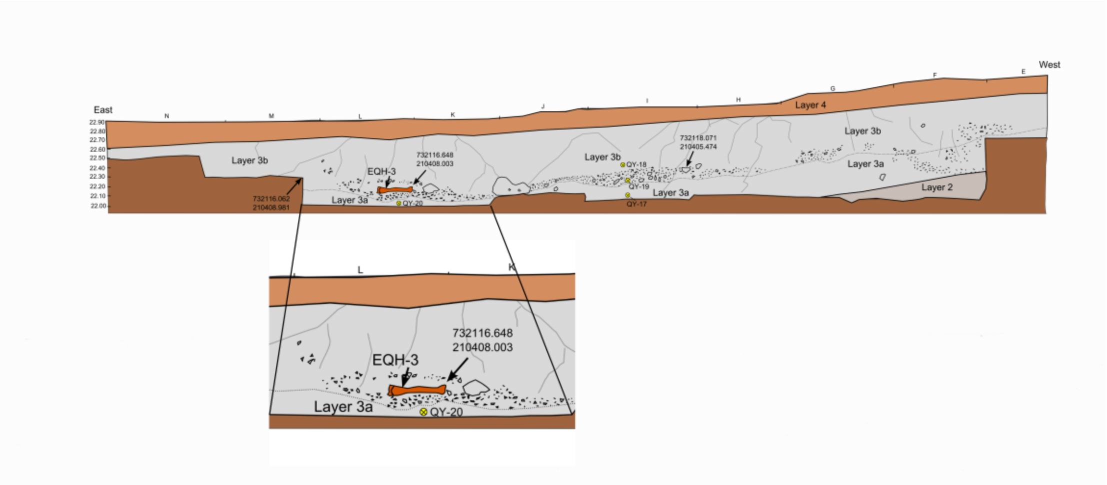

SI 1 Fig. 4. A section showing the sedimentological context of EQH-3 (top), and a close-up of its stratigraphic position (inset at bottom).

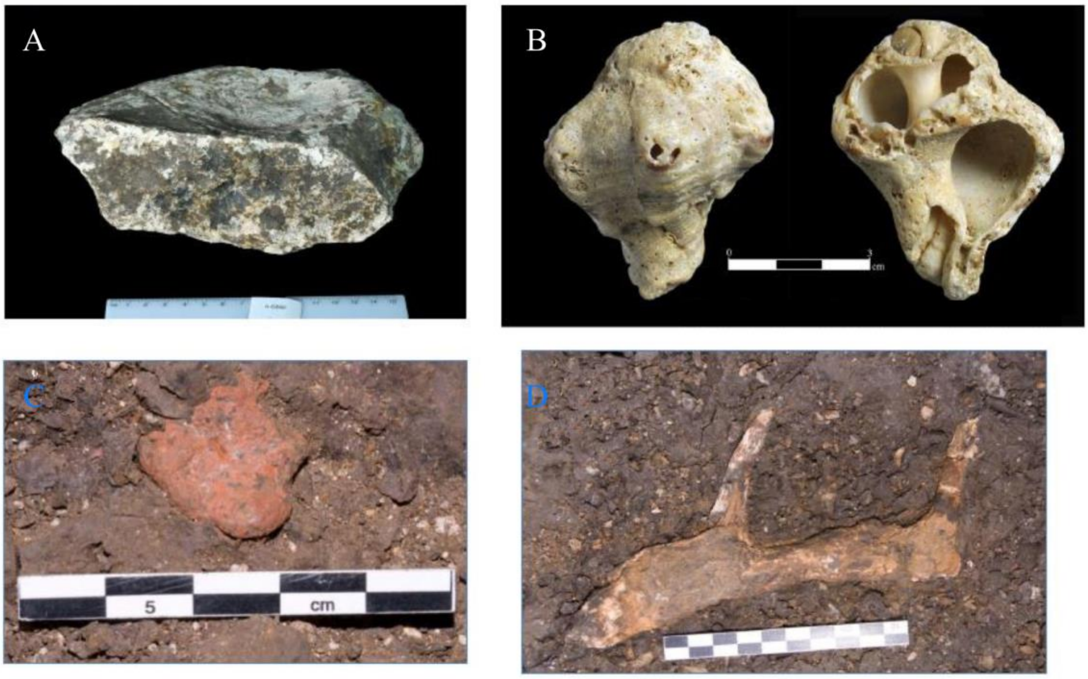

SI 1 Fig. 5. Finds from Layer 3b in Area B. (A) Stone manuport. (B) *Hexaplex trunculus* (Linnaeus, 1758) shell. (C) Ochre. (D) An antler of a roe deer.

Photo (A, B) Clara Amit, Courtesy of the Israel Antiquities Authority

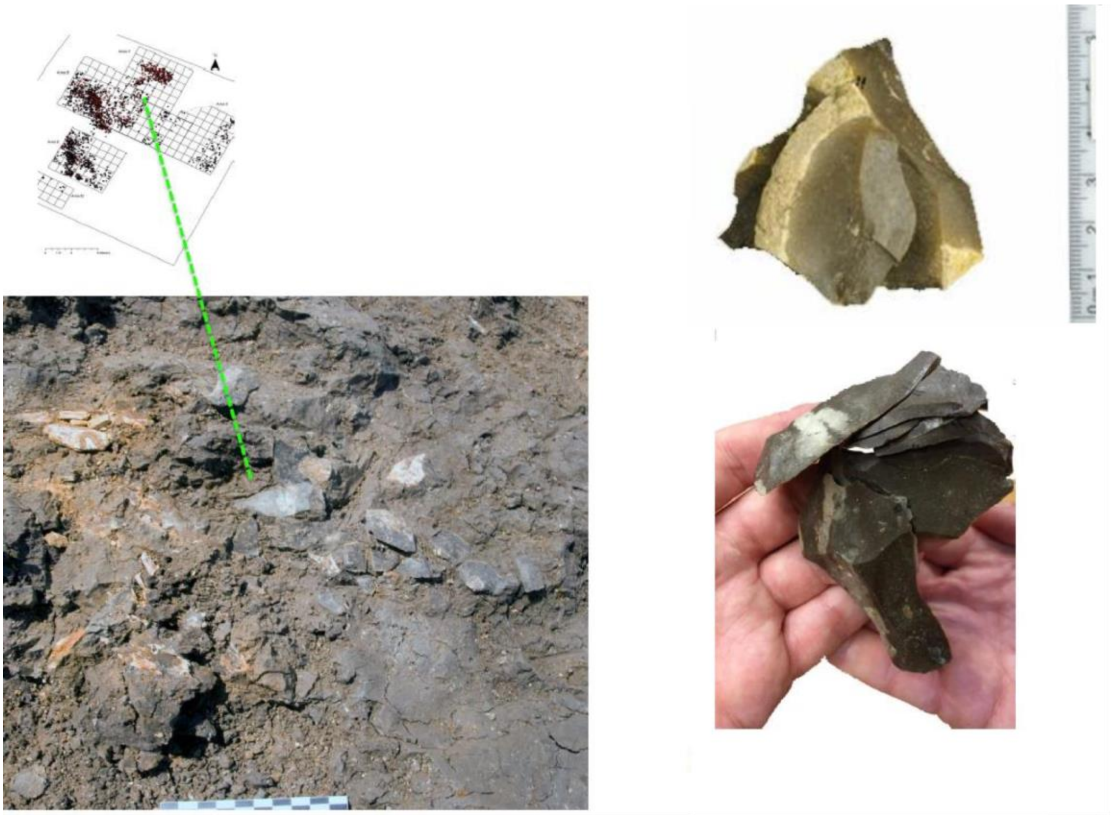

SI 1 Fig. 6. Refitted aggregates from Area B. Refits are laterally and vertically clustered (lower right).

Photo (Top right) Clara Amit, Courtesy of the Israel Antiquities Authority

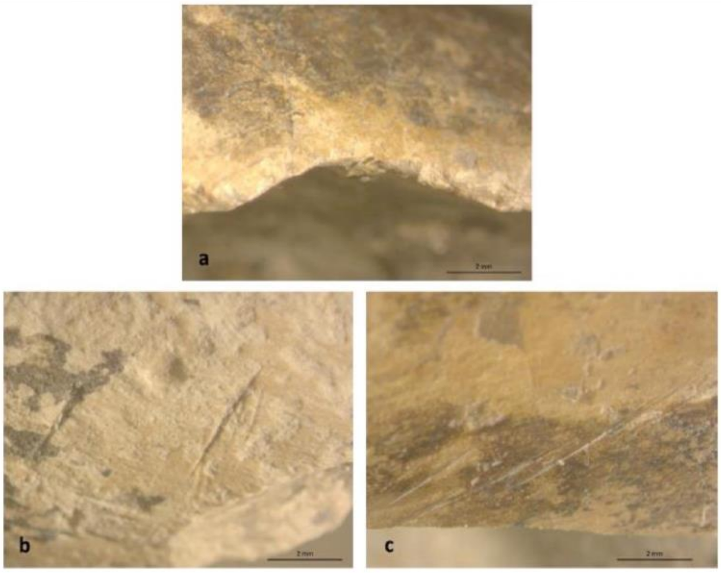

SI 1 Fig. 7. Examples of butchery at ‘Ein Qashish. (a) Medium ungulate (*Dama mesopotamica*) humerus (#2309) bearing hammerstone percussion signs (the conchoidal notch). (b) Large ungulate (*Bos primigenius*) radius (#1063) bearing filleting marks. (c) Medium ungulate (*Dama mesopotamica*) tibia (#1731) bearing filleting marks.

SI 2. Optically Stimulated Luminescence (OSL) Dating

The site sequence of the 2013 excavation was dated through optically stimulated luminescence. After site stratigraphy was established, samples were collected from freshly cleaned sections in the different excavation areas. We collected the samples under cover to prevent any exposure to sunlight and then stored them immediately in black, light-tight bags. We took a complementary sample from the same location for dose rate measurements. Several samples were collected from each of the stratigraphic layers exposed in the different sections.

Quartz in the range of 88-125 μm was extracted and measured under suitable dim orange light using routine laboratory procedures (71). After the sediment sample was sieved to the selected grain size, carbonates were dissolved by soaking in 8% HCl followed by rinsing and drying. Heavy minerals and most feldspars were removed using the Frantz magnetic separator, and HF (40%) etching for 40 min was used to dissolve the remaining feldspars and etch the quartz. The samples were then rinsed in 16% HCl overnight to dissolve any fluorides that may have precipitated.

We measured equivalent doses (D_e) for each sample on 17 to 23 aliquots prepared with 1 or 2 mm masks, using a modified single aliquot regenerative (SAR) protocol (72), and we calculated averages and errors using the central age model (CAM) (73). Measurements were carried out on Risø TL/OSL readers (models DA-12 or DA-15). Dose recovery tests over a range of preheats showed that a dose recovery of 100% can be obtained using a preheat of 10 s at 260°C, a test dose of ~9.3 Gy, and a test dose preheat of 5 s at 240°C. These measurement conditions were used throughout.

Alpha, beta, and gamma dose rates were calculated from the concentrations of the radioactive elements measured by inductively coupled plasma (ICP mass spectrometry (U and Th) or ICP atomic emission spectroscopy (K), using attenuation factors from (74). Cosmic dose rates were estimated from current burial depths, and water content was measured immediately after sampling (SI 2 Table 1).

To identify the most dominant age components in samples with scattered D_e values, we measured single grains from several samples. The data was processed and reliable grains were selected using criteria as in (75). The main age component was isolated using the finite mixture model (FMM) (74).

All samples show good performance with respect to OSL properties and D_e measurements: the OSL signal was bright and decayed rapidly to background levels, indicating a dominant fast component. Recycling ratios were within 8% of unity, indicating that the SAR protocol corrects appropriately for sensitivity changes, and IR signals were negligible. Dose distributions were mostly normal, with overdispersion values (an indication of scatter beyond that expected from the physical measurements) usually less than 25%.

The ages ranged from 9 ka at the top of the sequence to 70–75 ka at the base of the exposed layers (SI 2 Table 1; Fig. 2*A*). Once preliminary ages were available, it became apparent that samples EQHD-42, EQHD-44, EQHD-46, and EQHD-47 did not conform to the stratigraphic order. (These samples are indicated in SI 2 Fig. 1 as open squares and highlighted in gray in SI 2 Table 1.) Repeated analysis was carried out to ascertain that the D_e values and dose rates were measured correctly. While the D_e values resembled those of nearby samples (from the same unit or section; SI 2 Table 1), the dose rates for three of the four outlier samples were either much higher

(sample EQHD-42) or much lower (samples EQHD-44 and EQHD-46) than the mean dose rate calculated from all samples, 1.55 ± 0.32 Gy/ka.

The lower dose rates could have been caused by dilution with a low-dose-rate mineral, such as calcium carbonate ($CaCO_3$). The higher dose rates could have resulted from high concentrations of clay or heavy minerals. The carbonate contents were thus measured for these outliers, and for 8 additional samples collected for OSL dating from several sections (SI 2 Table 1). The carbonate content for most samples turned out to be in the range of 1–5%, whereas for one of the outlier samples (EQHD-44) it was 43.8%. Thus, the high carbonate content could explain the low dose rate of that particular sample. Since the resulting age is overestimated, dose rates might have been lowered in more recent times by the deposition of carbonates, and current dose rates do not represent the time-averaged dose rate for this sample over its geological history. However, the deposition of carbonates could not be the reason for the low dose rate in two additional samples, as their carbonate content is not high.

We also wanted to check whether the ages calculated for these samples using the site-averaged dose rate are more concordant and agree better with the stratigraphy. SI 2 Table 2 lists these outlying samples, showing their OSL age against the age expected from nearby samples (either from the same unit in other sections, or from the over- and underlying samples). The ages for these samples were recalculated using the averaged dose rates for the entire site (1.55 Gy/ka). For all four samples, the recalculated ages agree much better with the expected ages, further indicating perturbation in the dose rates of individual samples in rather recent times.

However, we did not find satisfactory clues for changes in dose rates over time for samples other than EQHD-44, and they were not included in further analyses.

SI 2 Fig. 1 shows the ages with their associated errors by stratigraphic layer. Aside from the outliers, the ages fall within range of 74 ka to 59 ka (solid squares). The stars indicate the stratigraphic locations of EQH-2 (a tooth) and EQH-3 (lower limb bones).

To obtain robust ages for individual layers and obtain a chronological framework for the site, ages from each layer across the sections were averaged, excluding the outliers; these averages are presented in Fig. 2*A*. SI 2 Table 3 lists the samples used to calculate the average for each layer, and its averaged age. Only one sample was collected from Layer 5b: sample EQHD-44 (marked with an asterisk), which turned out to be one of the outliers. As this was the only sample whose low dose rate could clearly be explained by a substantial addition of carbonates at a late stage in the sample's history, here we used the age calculated from the site-averaged dose rate (SI 2 Table 2).

Note the robust ages calculated for Layer 3a, for Layer 3b (where the hominin remains of EQH-3 were found), and for Layer 5a (where the tooth specimen EQH-2 was found). These ages bracket the time of the human remains to 66–68 ka. Note also that OSL dating cannot distinguish clearly between the ages of the lowermost and uppermost Middle Palaeolithic layers (1–5), and it appears that the sediments were deposited rapidly.

.

SI 2 Tables and Figures

SI 2 Table 1. Field and laboratory data for OSL samples with ages.

Lab code	Field number	Layer	Area	Depth (m)	Water content (%)	K (%)	U (ppm)	Th (ppm)	Ext. α (μGy/a)	Ext. β (μGy/a)	Ext. γ (μGy/a)	Cosmic (μGy/a)	Total dose (μGy/a)	No. aliquots	OD (%)	D_e (Gy)	CAM Age (Ka)	$CaCO_3$ (%)
EQHD-12	EQ5-U5	5a	T-EQH5	2.5	12.3	0.30	1.2	4.5	6	415	368	154	944±28	22/22	32	61±3	**65±3**	
EQHD-13	EQ5-U4	4	T-EQH5	3.0	35.8	1.08	2.3	12.6	11	953	796	145	1905±52	20/20	17	126±6	**66±3**	
EQHD-14<br>SG	EQ5-U3	3a	T-EQH5	4.0	26.9	0.55	2.1	9.3	10	671	622	129	1433±38	19/19<br>59/140	17<br>34	104±4<br>94±3	72±4<br>**66±3**	
EQHD-15	EQ5-U2	2	T-EQH5	4.5	26.6	0.55	2.3	8.8	10	682	621	122	1435±41	20/20	8	110±3	**76±3**	
EQHD-16<br>SG	EQ5-U1	1	T-EQH5	5.4	28.1	0.60	3.2	9.4	12	801	724	111	1648±49	21/23<br>3/4 42%	34<br>40	112±5<br>117±5	68±4<br>**71±4**	
EQHD-40	QY-3a	3a	F	4.5	27.2	0.62	2.3	9.9	10	730	668	122	1531±49	17/17	10	95±3	**62±3**	2.9
EQHD-41	QY-3b	3b	F	3.9	25.3	0.65	2.7	11.0	12	820	761	131	1724±56	17/17	14	102±4	**59±3**	1.0
EQHD-42	QY-4	4	F	3.6	25	1.16	2.6	14.0	13	1144	962	135	2255±55	16/17	11	101±3	**45±2**	0.3

Lab code	Field number	Layer	Area	Depth (m)	Water content (%)	K (%)	U (ppm)	Th (ppm)	Ext. α (μGy/a)	Ext. β (μGy/a)	Ext. γ (μGy/a)	Cosmic (μGy/a)	Total dose (μGy/a)	No. aliquots	OD (%)	D_e (Gy)	CAM Age (Ka)	$CaCO_3$ (%)
EQHD-43	QY-5a	5a	F	2.4	24.7	0.60	2.0	10.1	10	713	662	156	1540±39	17/17	18	103±5	**67±4**	2.1
EQHD-44	QY-5b	5b	F	1.9	24.6	0.35	1.35	5.6	6	429	392	166	993±23	16/17	26	83±5	**83±5**	43.8
EQHD-45	QY-6	6	F	1.4	15	0.56	1.6	7.9	9	675	592	176	1452±58	17/17	39	14±1	**9±1**	21.3
EQHD-46	QY-15	4	E	3.4	25	0.65	0.7	3.8	4	492	325	139	959±25	15/17	40	105±5	**109±6**	4.1
EQHD-47	QY-16	5a	E	2.3	24.6	0.62	1.9	8.9	9	692	614	158	1473±47	17/17	25	124±8	**84±6**	0.6
EQHD-48	QY-17	3a	E	4.1	22.2	0.60	1.9	10.0	10	716	663	128	1516±54	17/17	11	113±4	**74±4**	
EQHD-49	QY-18	3b	B	3.6	22.3	0.66	1.9	10.8	10	764	704	135	1614±41	17/17	15	105±4	**65±3**	2.1
EQHD-50<br>SG	QY-19	3a	B	3.9	14.8	0.42	1.6	6.7	8	567	509	131	1209±26	17/17<br>2/3 57%	13<br>30	116±4<br>85±2	96±4<br>**71±3**	38.6
EQHD-51	QY-20	3a	B	4.2	26.9	0.59	2.3	11.6	11	753	731	127	1621±54	17/17	10	113±3	**70±3**	2.2
EQHD-52	QY-14	4a	E	3.8	26.3	1.25	2.3	11.5	11	1099	850	132	2029±63	17/17	11	151±5	**72±3**	1.0

No. aliquots – the number of aliquots used for the average D_e out of those measured. For single grains (SG), this indicates the number of components isolated using the finite mixture model (FMM) (Galbraith & Roberts 2012) and the percentage of the largest component. OD, overdispersion. D_e averages and errors were calculated using the central age model (CAM). Ages of single-grain samples, used in the remainder of the discussion, are in **bold.** Samples highlighted in gray were not used for layer averages.

SI 2 Table 2. Samples that were not in stratigraphic order and their recalculated ages using a site-averaged dose rate of 1.55 Gy/ka. (See text for details.)

Sample	D_e (Gy)	Dose rate (Gy/ka)	Age (ka)	Expected Age (ka)	Recalculated Age (ka)	$CaCO_3$ (%)
EQHD-42	101	2.26	45±2	<~60-65	65	0.3
EQHD-44	85	0.99	83±5	<~60-65	54	43.8
EQHD-46	105	0.96	109±6	<~70	68	4.1
EQHD-47	124	1.47	84±6	<~70	80	0.6

SI 2 Table 3. Averaged ages for each unit. The ages for Layer 3b are shown from above and below the femur; an average for the entire unit is also shown.

Layer	Sample lab codes (EQHD)	N	Average (ka)
1	16	1	71±4
2	15	1	76±3
3a	14,40,48,51	4	68±5
3b (below femur)	41,50	2	65±8

Layer	Sample lab codes (EQHD)	N	Average (ka)
3b (above femur)	49	1	65±3
3b (average)	41,49,50	3	65±6
4, 4a	13, 52	2	69±3
5a	12,43	2	66±1
5b	44	1	54±5*
6	45	1	9±1

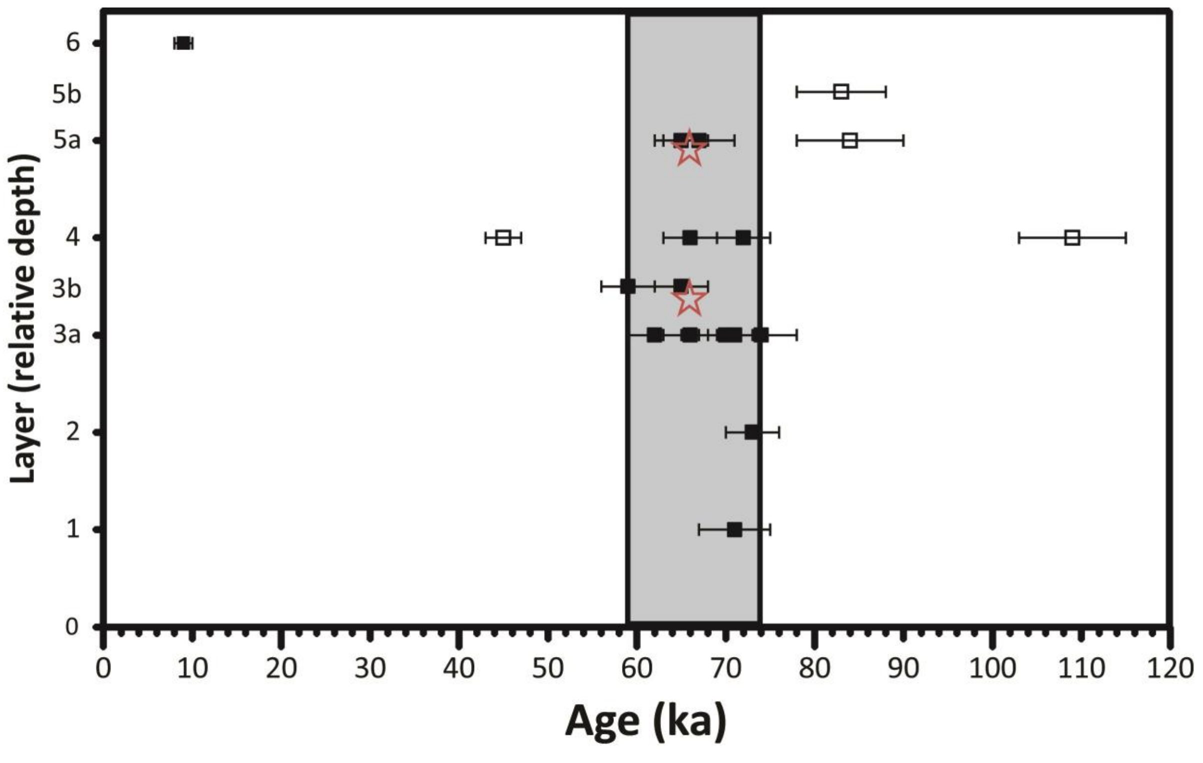

SI 2 Fig. 1. Ages of the stratigraphic layers (black rectangles) with their associated errors (bars). Red stars indicate human remains. Open squares indicate the outliers.

SI 3. EQH-2 (Upper Third Molar)

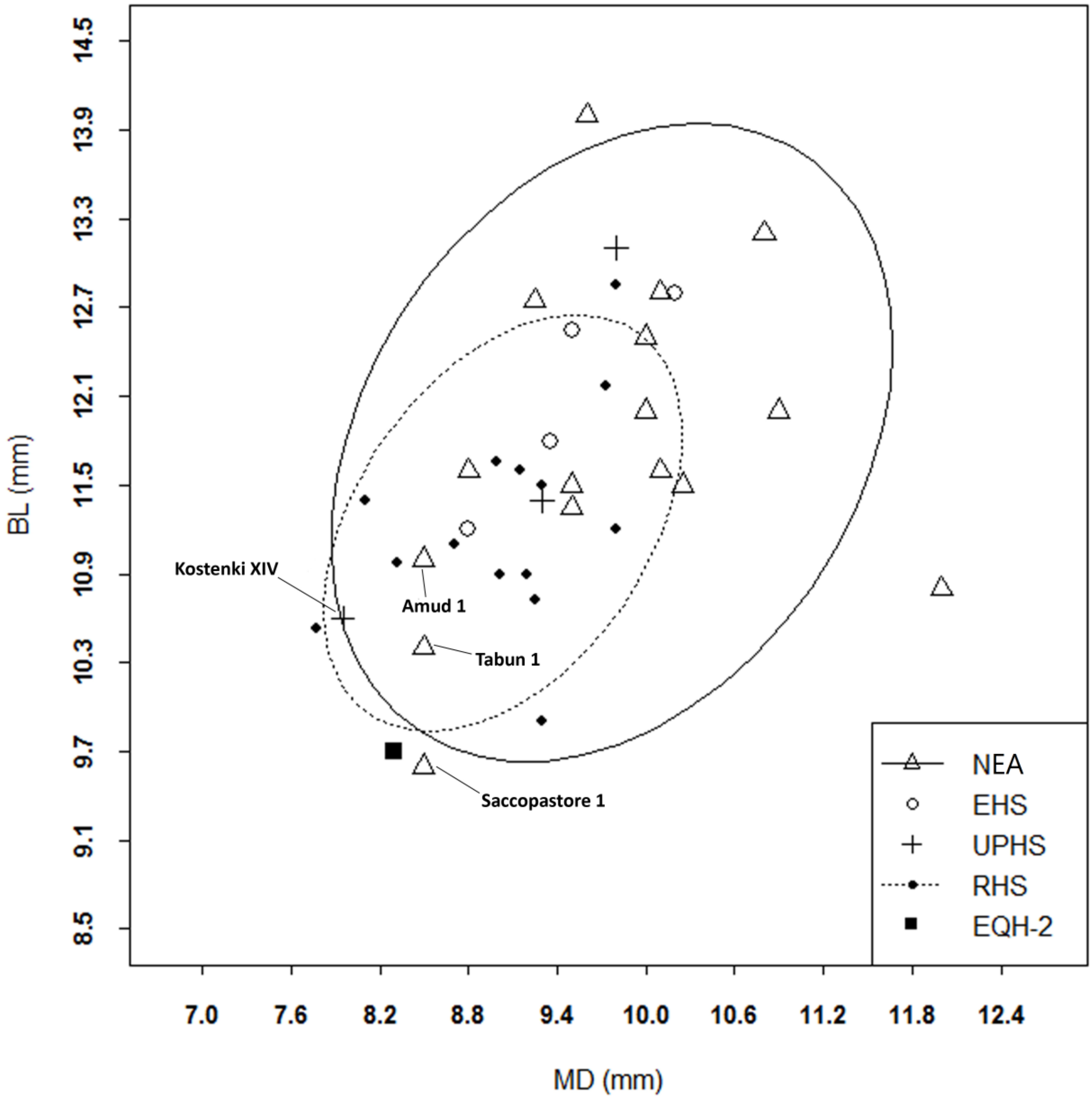

SI 3 Fig.1. Scatterplot of MD diameter vs. BL diameter of M^3. NEA, Neandertal; EHS, early *H. sapiens*; UPHS, Upper Palaeolithic *H. sapiens*; RHS, recent *H. sapiens*. In the scatterplot, the 95% confidence ellipses are reported for NEA and RHS.

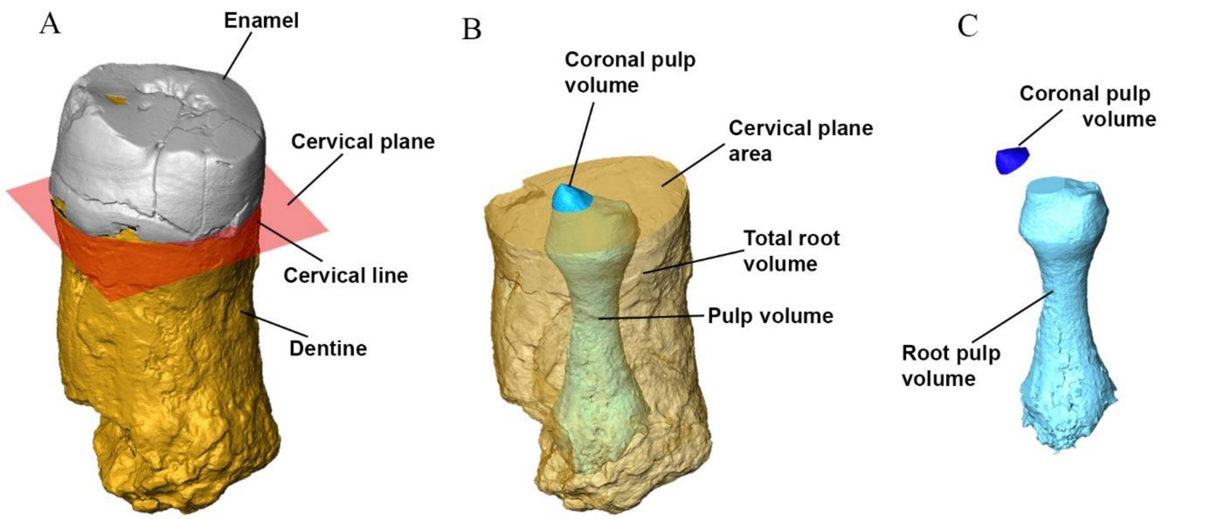

SI 3 Fig. 2. Root metric analysis. (A) The root of EQH-2 (RM^3**)** was separated from the crown along the best-fit plane computed at the cervical line (cervical plane). (B) The root was separated in two parts: total root volume and pulp volume. (C) The pulp volume was divided by the cervical plane into coronal pulp volume and root pulp volume.

SI 3 Table 1. Nonmetric dental traits observed in the enamel-dentine junction of EQH-2 (RM^3) vs. the frequency of these traits (%) in Neandertals (NEA), early and Upper Palaeolithic *H. sapiens* (EHS and UPHS), and recent *H. sapiens* (RHS).

Specimen/Taxon	*n*	Hypocone	Carabelli's trait	Distal accessory tubercle	Mesial accessory tubercle	Paracone accessory cusp
EQH-2	1	Absent	Absent	Absent	Present	Present
NEA	16	100	56.6	31.25	50	100
EHS and UPHS	12	83.3	41.5	16.6	50	41.6
RHS	18	83.3	61.1	11.1	11.1	38.8

SI 3 Table 2. Dental crown diameters (MD and BL), in mm, of M^3 in EQH-2 and the comparative sample.

		MD				BL			
Specimen/Taxon	*n*	Mean	SD	Min.	Max.	Mean	SD	Min.	Max.
EQH-2	1	8.3				9.7			
NEA	16	9.76	0.96	8.5	12	11.78	1.10	9.6	13.2
EHS	4	9.46	0.57	8.8	10.2	12.08	0.72	11.2	12.8
UPHS	3	9.01	0.95	7.95	9.8	11.7	1.27	10.6	13.1
RHS	14	9.03	0.61	7.77	9.8	11.24	0.71	9.9	12.85

NEA, Neandertal; EHS, early *H. sapiens*; UPHS, Upper Palaeolithic *H. sapiens*; RHS, recent *H. sapiens*. Neandertals: Jersey 1, La Quina 5, Le Moustier 1, Saccopastore 2, Spy 1, Spy 2, Tabun 1, Vergisson 1, Vergisson 2, La Croze del Dua 3, La Croze del Dua 4 (76), Amud 1 (77), Shanidar 1, Shanidar 2, Shanidar 4 (33), Krapina 58 (78). EHS: Skhul 4, Skhul 5, Skhul 7 (76), Qafzeh 9 (79). UPHS: Dolni Věstonice 3 (76), Kostenki XIV, Sungir 2 (provided by Bence Viola, Institute of Anthropology, Russian Academy of Sciences, Saint Petersburg). RHS: Vasilyevka III-55 Sloi IV 12, Vasilyevka III-55 N 33, NHMW Breitinger Nr. 87, NHMW Breitinger Nr. 85, NHMW 811, NHMW 9687, Bruckneudorf G905/1, Bruckneudorf G899, NHMW 6034, Bystrovka 3 K7 N 30, NHMW 6031, NHMW 15358, NHMW 15357, Ushauz Cave Sk 1 (provided by Bence Viola, Institute of Anthropology, Russian Academy of Sciences, Saint Petersburg).

SI 3 Table 3. List of fossil and extant human upper third molars (M3) used for enamel thickness and root analysis.

Taxon	Specimen	Wear stage[1]	Enamel thickness	Root
NEA	BD8	2	X	
	El Sidron SD332	2	X	
	El Sidron SD621	1	X	X
	El Sidron SD741	2	X	
	Kebara KMH24	2	X	
	Krp D162	3	X	X
	Krp D163	2	X	X
	Krp D170	2	X	
	Krp D173	3		X

Taxon	Specimen	Wear stage[1]	Enamel thickness	Root
	Krp D178	2	X	X
	Krp D180	2	X	
	Krp D58	2	X	X
	Krp D99	2	X	
	Le Moustier 1	1	X	
	Marillac	1	X	X
	St-Cesaire	2	X	X
EHS	Qafzeh 11	1	X	
	Qafzeh 26	2	X	X
	Skhul 4	4	X	
UPHS	Combe Capelle	2	X	X
	Equus Cave EQ-H12	4	X	X
	Hayonim 19	1	X	
	Hayonim 25	3	X	X
	Hayonim 8	3	X	X
	Les Rois	4		X
	Nahal Oren 24	1	X	X
	Nahal Oren 16	2		X
	Oberkassel D999	2	X	X
	Ohalo H2	2	X	
	Villabruna 1	1	X	X
RHS	565	2	X	X
	FJRI r2064-1420	1	X	
	FJRI r2643-1941	1	X	
	FJRI r605-1185	2	X	X
	M072	2	X	X
	M091	2	X	X
	M152	2	X	X
	M157	2	X	X
	M160	2	X	
	M194 30	2	X	X
	M197	2	X	X
	M199	2	X	X

Taxon	Specimen	Wear stage[1]	Enamel thickness	Root
	M20	2	X	X
	M246	2	X	X
	M249	2	X	X
	M65 0385	2	X	X
	Ulac 536	2	X	X
	Wittenberg 2192-39-a	2	X	X

NEA, Neandertal; EHS, early *H. sapiens*; UPHS, Upper Palaeolithic *H. sapiens*; RHS, recent *H. sapiens.*

[1]Based on Molnar (30).

SI 3 Table 4. Three-dimensional enamel thickness of EQH-2 (RM^3) standardized to z scores (for RET index) of the *H. heidelbergensis* (HH), Neandertal (NEA), early and Upper Palaeolithic *H. sapiens* (EHS and UPHS), and recent *H. sapiens* (RHS) M^3 sample for different wear stages. (Standard deviation is indicated in brackets.)

Specimen/Taxon	Wear stage[1]	*n*	AET (mm)		RET (scale free)		z scores for RET index
			Mean	Range	Mean	Range	
EQH-2	3		0.95		18.9		
HH	2	1	1.2		23.5		
NEA	1–3	16	1.22 (0.13)	0.95–1.45	19.66 (2.25)	15.42–28.36	-0.33
EHS and UPHS	1–3	10	1.39 (0.17)	1.05–1.58	25.01 (3.85)	19.42–32.37	-1.58
EHS and UPHS	4	2	0.88 (0.02)	0.87–0.90	15.49 (1.28)	14.58–16.40	1.01
RHS	1/2	18	1.45 (0.14)	1.23–1.70	26.48 (4.20)	20.19–35.92	1.81

AET, average enamel thickness index; RET, relative enamel thickness index.

[1]Based on Molnar (30).

SI 3 Table 5. Three-dimensional root analysis of EQH-2 (RM³) standardized to z scores of the *H. heidelbergensis* (HH), Neandertal (NEA), early *H. sapiens (*EHS), Upper Palaeolithic *H. sapiens (*UPHS), and recent *H. sapiens* (RHS) M³ sample.

Specimen/ Taxon	*n*	R.L.		T.R.V.		P.V.		C.P.V.		R.P.V.		C.P.A.	
		Mean (SD)	z score	Mean (SD)	z score	Mean (SD)	z score	Mean (SD)	z score	Mean (SD)	z score	Mean (SD)	z score
EQH-2	1	14.24		600.14		73.39		1.00		72.38		55.27	
HH	1	13.12		388.93		30.75		2.64		28.11		52.74	
NEA	7	15.77 (1.90)	0.80	728.61 (200.06)	0.64	65.89 (27.25)	0.27	3.36 (3.43)	0.68	62.62 (28.74)	0.33	80.34 (7.42)	3.37
EHS	1	14.20		483.34		57.25		2.09		55.15		67.40	
UPHS	9	12.89 (1.63)	0.82	381.45 (78.18)	2.79	27.82 (8.073)	5.64	3.13 (4.72)	0.33	24.68 (5.70)	8.36	66.48 (7.14)	1.57
RHS	15	11.44 (1.73)	1.62	393.57 (111.73)	1.84	38.15 (17.26)	2.04	4.64 (5.34)	0.68	32.63 (13.96)	2.84	60.85 (11.42)	0.48

R.L., root length; T.R.V., total root volume; P.V., pulp volume; C.P.V., coronal pulp volume; R.P.V., root pulp volume; C.P.A., cervical plane area.

SI 4. Description of the EQH-3 Bones

The EQH-3 specimen consists of five lower limb bones: left femur, right and left tibia, and right and left fibulae. Only the femur and tibiae are the subject of this research.

The femur (Figure 4)

Nearly complete from the articular condyles up to the head, the femur is eroded at the epiphyses and fragmented at the diaphysis. It is robust and highly curved in the sagittal plane, with the apex of the curvature distal to the midshaft. The bicondylar length of the femur is 438 mm and its biomechanical length is 407 mm, both within the range reported for *H. sapiens* and slightly higher than the mean for Neandertals. Note that the bicondylar length might have been slightly longer if the bone were complete (SI 4 Tables 1 and 3). Broken parts of the bone were reconstructed by Yoel Rak.

The proximal femur: The femoral head is partially broken and eroded. The neck is eroded, and its remaining part is distorted, probably due to taphonomic changes. There are remains of the greater and lesser trochanters, but they do not suffice for a full description. The neck shaft angle (119°) indicates coxa vara. The angle of torsion (19°) indicates a high degree of anteversion (SI 4 Tables 1 and 3).

The femoral shaft: The shaft is complete but fragmented. Its overall shape is robust and highly curved, with a smooth anterior surface, while the posterior surface is roughened by the linea aspera. The femoral pilaster (linea aspera pilaster) on the posterior surface of the shaft is missing. The popliteal surface on the posteroinferior part of the femoral shaft is flat and mediolaterally broad. The midshaft shape ratio (with a pilastric index of 99.1) indicates a rounded cross section (the anteroposterior diameter and the mediolateral diameter are nearly equal). The midshaft robusticity index (14.9) is very high in comparison with that of *H. sapiens* (early and recent) and Neandertals, indicating a very robust femur (SI 4 Table 3). The

subpilastric index of the femur (85.6%) reflects the widening of the femoral shaft toward its distal end (at 25% of the femur length).

The distal femur: Both the medial and the lateral condyles of the femur are preserved, but the medial condyle is eroded on its medial side. The medial articular surface is nearly complete; its surface is smooth and wide. The medial epicondyle is eroded, but the adductor tubercle projects medially from the proximal end of the medial condyle. The lateral condyle is longer (along the anteroposterior axis) and narrower (along the mediolateral axis) than the medial condyle. A shallow pit (3 cm in diameter) can be seen on the lateral surface of the lateral condyle, probably due to postmortem pressure exerted on the bone. The intercondylar fossa, located between the two distal articular condyles, is extremely narrow. Its unique shape is not usually seen in the distal femora of *H. sapiens* or Neandertals. The maximum mediolateral condylar breadth and the epiphyseal breadth ratio are small compared with those of Neandertals and *H. sapiens* (SI 4 Tables 1 and 3). This is the result of the narrow lateral condyle and the very narrow intercondylar notch.

Computed tomography (CT) scanning of the bone reveals that the total cross-sectional area of the midshaft is large and the cortical bone is relatively thick (SI 4 Table 3). The CT scan also shows the presence of an epiphyseal line at the distal femur, in accordance with ossification stage 3 out of 4, where the fourth stage implies full ossification (41, 42).

The right tibia (Fig. 4*F*)

The right tibia is missing its proximal part: the remains include the diaphysis, distal to the soleal line, and the distal epiphyses. The proximal end of the tibial shaft is eroded, and the shaft itself is fragmented and slightly distorted. The anterior crest of the tibial shaft is rounded. The interosseous border is smooth and rounded, resulting in a continuation between the posterior, the medial, and the lateral surface of the tibial shaft. The medial surface of the tibia is wide, with a fracture line running along its long axis. Just proximal to the talar facet is

a marked groove running from the medial to the lateral surface, on the anterior side. This groove was caused by tools used in the excavation.

The distal end of the right tibia is remarkably complete. The fibular notch, on its lateral side, is flat and wide. The talar facet has a trapezoid shape; its lateral side is wider than its medial one. The prominent medial malleolus is nearly complete. A squatting facet is seen on the anteroinferior edge of the tibia (SI 4 Tables 2 and 4).

The left tibia (Fig. 4*E*):

The left tibia is nearly complete; it extends from the tibial plateau to the talar facet, but it is missing the medial malleolus. The tibial plateau is flat, and its peripheral edges are eroded. A robust intercondylar tubercle (the medial part of the intercondylar eminence) is clearly visible between the two condyles. The lateral part of the intercondylar eminence is missing. Three centimeters distal to the tibial plateau, on the anterior surface, there is a prominent tibial tuberosity. The superior fibular articular facet is clearly visible on the posterolateral side of the lateral condyle. When viewed laterally, the shaft of the left tibia is fragmented and slightly curved. The shaft is noticeably flattened mediolaterally (i.e., platycnemic). As in the right tibia, the anterior crest of the tibial shaft is rounded, and the interosseous border is smooth and rounded, forming a continuation between the posterior and the lateral surface of the tibial shaft. (Similar morphology is seen in the tibiae of Palomas 96, Shanidar 1, and Shanidar 2 (80)). The medial surface of the EQH-3 tibia is wide, with a fracture line running along the middle of the surface from the proximal to the distal end. Between the proximal and middle thirds of the posterior surface, the soleal line can be seen (SI 4 Tables 2 and 4).

CT scanning of the tibiae reveals the presence of an epiphyseal line at the proximal and distal tibia. This implies ossification stage 3 out of 4 (where stage 4 indicates complete ossification) (41, 42).

Discussion

Neandertal vs. early *H. sapiens*

We attributed EQH-3 to a Neandertal on the basis of femoral and tibial characteristics. Two hominin species are known to have lived in the area (northern Israel) at the MP: early *H. sapiens* and Neandertals. It has been well established that the Neandertal femora have a suite of characteristic features that include a thick, rounded shaft with a very little or no femoral pilaster, a small angle between the neck and the shaft (coxa vara), and a highly robust femoral shaft (31–34). Researchers have also recently emphasized the high curvature of the femoral shaft of Neandertals as compared to that of *H. sapiens,* and the high cortical bone percentage in the femoral midshaft (35, 36, 81). A number of reasons were suggested for this characteristic morphology, including elevated activity level, adaptation to cold, and locomotion (58, 82–84). The characteristic features documented for the femora of early *H. sapiens* include a gracile and straight femoral shaft, with a small articulation area relative to shaft length. The neck shaft angle is high (coxa valga) (21, 36, 79). The midshaft shows a drop-like cross section (long anteroposterior diameter and short mediolateral diameter), with a well-defined femoral pilaster.

The EQH-3 femur is very robust, and the midshaft robusticity index (14.9) is much higher than that of *H. sapiens* (early and recent) and Neandertals; the index is similar to that of the highly robust femora from Shanidar (33). The midshaft cross section (pilastric index, 99.1%) is rounded (the anteroposterior diameter and the mediolateral diameter are nearly equal), a well-documented Neandertal characteristic that contrasts with the more oval shape of the midshaft in *H. sapiens*, due to a more elongated anteroposterior diameter in *H. sapiens* (33, 36). The femoral shaft of EQH-3 lacks a pilaster and shows a high degree of curvature; both are well-documented Neandertal features. The neck shaft angle (119°, coxa vara) is close to the Neandertal mean and smaller than in *H. sapiens* (124°–130°). The midshaft cross

section of EQH-3 reveals a large cross-sectional area with a relatively high percentage of cortical bone. All of the above characteristics suggest that the femur of EQH-3 belonged to a Neandertal rather than to early or recent *H. sapiens*. It should be noted, however, that Neandertals are characterized by a large articular area in relation to femur length, while in *H. sapiens*, the articular area is smaller (84). The relatively small articular area of the EQH-3 femur (epiphyseal breadth ratio = 16.9) is at the lower end known for Neandertals (16.8 for Tabun C1) and below the Neandertal mean (18.9 ± 1.4) (21, 36). The epiphyseal breadth ratio of EQH-3 is close to that of *H. sapiens* (17.1 ± 1.2).

The Neandertal tibia is robust and characterized by an almond-shape cross section with a relatively rounded anterior crest, almost no prominence of the lateral interosseus crest, and a rounded posterior margin (38). The tibia thus contrasts with the more gracile tibiae of early and recent *H. sapiens,* and their generally angular anterior and interosseous crests (38, 86). The Neandertal tibial diaphysis has been described (33, 86, 87) as mesocnemic or euricnemic (average or wide mediolaterally) while that of *H. sapiens* has been described as platycnemic (narrow mediolaterally), mesocnemic, or euricnemic. It is worth noting that the tibia of early *H. sapiens* is very wide mediolaterally. The tibial tuberosity of Neandertals projects more anteriorly than in *H. sapiens*, and the tibial condyles are in a posterior position. The tibiae of EQH-3 are morphologically similar to Neandertal tibiae in the former's distinctive, rounded anterior crest and interosseous crest. The robusticity index of the EQH-3 tibia is higher than that of *H. sapiens* and similar to that of Neandertals (SI 4 Table 4). However, the tibial diaphysis is mediolaterally narrow (platycnemic) in comparison to its anteroposterior length, a feature known to occur in recent *H. sapiens* and not common in Neandertals or in early *H. sapiens*. It is worth noting that the tibia of another Neandertal from Israel, Amud 1, is also platycnemic, with a cnemic index of 16.5, similar to that of EQH-3.

The crural index in Neandertals is smaller than that of *H. sapiens*, indicating that the distal segment (tibia) is short in relation to the proximal segment (femur). The crural index of

EQH-3 is lower than the *H. sapiens* mean and higher than the Neandertal mean. The combination of the femoral and tibial features, together with the low crural index, indicates that the bones belonged to a Neandertal.

Age estimation

We used three long-bone indicators to estimate the age of EQH-3: stage of epiphyseal union, bone length, and age-related pathology (osteoarthritis).

Stage of epiphyseal union: We used the five stages of epiphyseal union described by O'Connor (41) for long bones: non-union (0); beginning union (1), when the epiphyseal and diaphyseal surfaces closely approximate each other; active union (2), when the epiphysis and diaphysis cap each other; recent union (3), when the epiphysis and diaphysis have united to form a single unit of bone, the position of the former epiphysis and diaphysis can still be distinguished, and a fine line of fusion of greater density may remain between the epiphysis and diaphysis; and complete union (4), when the epiphysis and diaphysis are united as a single unit of bone. The distal epiphysis of the femur of EQH-3 and the proximal and distal epiphysis of the tibiae can be assigned to stage 3. A fine line of fusion of greater density (epiphyseal line) is clearly visible in the CT scan between the epiphysis and diaphysis. Stage 3 can be seen in young adults between the ages of 15 and 22 in modern human populations.

Long bone length: We compared the length of the bones of EQH-3 to the femoral and tibial lengths of other Neandertals. The comparison indicates that the long bones of EQH-3 reached full or nearly full adult length.

Age-related pathology: No age-related pathology was identified on any of the bones. We can conclude from these three indicators that the bones of EQH-3 belonged to a young adult (15–22 y) (41, 42).

Male vs. female

The pattern of sexual dimorphism in European and Near Eastern Neandertals is virtually indistinguishable from that of recent *H. sapiens.* Male and female Neandertals are distinguished by their limb bone lengths and articular dimensions. The degrees of size dimorphism found in single-site samples and in the total sexable Neandertal sample are within the expected ranges of variation for the recent *H. sapiens* samples. Furthermore, no differences exist between the Neandertal and recent *H. sapiens* samples with respect to postcranial robusticity as an indicator of sexual dimorphism. In both the fossil and recent samples, the males tend to be slightly more robust than the females, and there is extensive overlap between the sexes (69).

In terms of length and robusticity, the femur of EQH-3 falls within or above the mean for Neandertal males. The tibia presents a somewhat more complex picture. The tibial length falls above the mean for Neandertal males and significantly above the length of Neandertal females. Tibial robusticity, however, falls closer to the mean for Neandertal females than Neandertal males (SI 4 Table 5). Neandertal males also show a lower cnemic index than Neandertal females (SI 4 Table 5); the platycnemic tibia of EQH-3 is at the lower range known for Neandertal males (similar to that of Amud 1) and significantly below the range for Neandertal females (33, 69).

We conclude from this analysis that the bones of EQH-3 belonged to a Neandertal male.

Estimated stature

Stature estimation is used in the reconstruction of the individual's physique during life and provides an indication of size and body mass (40). In fossil hominins, stature is estimated using correlations from recent *H. sapiens* populations. Many formulas for using long bones to calculate stature are found in the literature (88-91, to name a few). We used three types of formulas: formulas based on femur length, formulas based on tibial length, and formulas based on femoral and tibial length (SI 4 Table 6). Using these formulas, we estimated the

stature of EQH-3 to be between 159. 9 and 168.6 cm, and we estimated the mean calculated height of EQH-3 to be 163.6 cm (SI 4 Table 6).

The stature of EQH-3 is within the normal range for modern humans, and the specimen would be classified as being of below medium height. The height of EQH-3 is significantly below the estimated height for early *H. sapiens* (males and females), close to the mean for Neandertal males, and above the mean for Neandertal females. A comparison with the hominin specimens from Sima de los Huesos shows that the stature of EQH-3 falls right in the middle between the males and the females (40) (SI 4 Table 7).

Discrepancy between shaft robustness and small articular area

The surprising combination of the very robust diaphysis and the slender epiphysis in the femur of EQH-3 gives rise to some intriguing hypotheses.

The morphology of articular surfaces reflects their weight-bearing properties as well as the stability and mobility of the joints to which they contribute. Osteogenic responses to mechanical loading are known for compact bone in the diaphysis cross section and for trabecular bone in the epiphysis (92). Lieberman (92) claims that the articular surface area is ontogenetically constrained and related to locomotor behavior at the species level and to body mass at the individual level. At the same time, diaphysis cross-sectional geometry is related to individual variations in activity level. This would suggest that EQH-3 was relatively lightweight or avoided bearing weight on his left knee while still maintaining a high activity level.

Knee pathology

The distal femur and proximal adjacent tibia (left) exhibit some morphological peculiarities. These include the very narrow intercondylar notch, the small lateral articular facet of the distal femur, and the prominent tibial intercondylar eminence between the two

tibial plateaus. These features are different from the femur and tibia of Neandertals and *H. sapiens* (SI 4 Tables 3 and 4). In an attempt to understand the nature of these features, we tested three hypotheses:

(1) This is a pleisiomorphic characteristic that can be found in the femur or tibia of other hominins.

(2) This morphology is the result of taphonomy.

(3) The combination of these morphologies represents knee pathology.

After examining the literature and casts of the distal femur and proximal tibia of both *H. erectus* specimens and australopithecines, we concluded that the unique morphology of the knee joint of EQH-3 is different from the morphology of the knee joints of *H. erectus* and australopithecine specimens. We therefore rejected our first hypothesis.

The narrow intercondylar notch might indeed be a result of taphonomical changes, but these cannot be the cause of the small articular condyles and the protruding intercondylar eminence. We therefore partially rejected our second hypothesis.

The medial intercondylar eminence is the attachment area for the anterior cruciate ligament (ACL), one of the four major ligaments that stabilize the knee joint (93). This ligament prevents the knee from going into hyperextension. A narrow intercondylar notch together with a very prominent intercondylar eminence is associated with a well-known knee pathology—a bony avulsion of the ACL (94–96). These injuries occur most commonly in skeletally immature individuals between the ages of 8 and 14 (43). The mechanism for ACL tearing with or without avulsion is usually traumatic: an unexpected knee hyperextension or a blow to the lateral side of the knee. Patients with ACL avulsion fractures will develop knee hemarthrosis within 12–16 hours, and an inability to walk and run in the days or weeks after the injury. In order to survive, EQH-3 would have had to rely on help from members of his group shortly after the injury occurred. After the acute stage is over, in the months and years after the injury, individuals with ACL avulsion fractures can walk and run but might suffer

from knee instability when trying to bear weight (94, 96). If such a pathology did occur in the knee of EQH-3, he might have suffered from instability of the left knee joint and thus tried to bear less weight on his left leg than on his right leg. The small articular surface of the distal femur might be the result of that pathology, as articular surface area is directly related to the amount of axial pressure exerted on the joint. We therefore determined that the third hypothesis is most likely the correct one.

SI 4 Table 1. Description of femur measurements. The numbering of the measurements corresponds to the numbers in SI 4 Fig. 1; note that the measurements in brackets are not depicted in the figure. Unless otherwise specified, measurements are in millimeters.

Measurement	Description
1. Femur bicondylar length (Martin* #2)	Maximum length between the femoral head and the distal condyles
2. Femur biomechanical length	The distance between the most inferior point of the superior femoral neck and the distal condyles
3. Neck shaft angle (degrees) (Martin #29)	The angle between the shaft and the neck
[4. Torsion angle (degrees) (Martin #28)]	The angle between the axis of the femoral neck and the tangent of the posterior surface of the femoral condyles
[5. Femoral AP midshaft diameter (Martin #6)]	The anteroposterior length of the femoral midshaft
6. Femoral ML midshaft diameter (Martin #7)	The mediolateral breadth of the femoral midshaft
[7. Midshaft shape ratio (pilastric index)]	(Anteroposterior midshaft diameter/mediolateral midshaft diameter) ×100
[8. Midshaft circumference (Martin #8)]	The minimum circumference of the femoral midshaft
[9. Subpilastric index]	(Anteroposterior diameter/mediolateral diameter) × 100, at the inferior quarter of the femoral shaft (75% of femur length)
[10. Robusticity index[†]]	(Mediolateral midshaft diameter/bicondylar breadth) × 100

Measurement	Description
[11. Robusticity index‡]	(Mediolateral midshaft diameter + anteroposterior midshaft diameter) × 100/femur bicondylar length
12. Maximum ML condylar breadth (Martin #21)	The maximum mediolateral breadth of the distal femur
13. ML breadth of lateral condyle	The maximum mediolateral breadth of the lateral condyle
[14. AP length of lateral condyle]	The maximum anteroposterior length of the lateral condyle
15. ML breadth of medial condyle	The maximum breadth of the medial condyle
16. AP length of medial condyle	The maximum anteroposterior length of the medial condyle
[17. Epiphyseal breadth ratio]	(Maximum condylar breadth/biomechanical length) × 100
18. Intercondylar fossa breadth	The distance between the medial wall of the lateral condyle and the lateral wall of the medial condyle at the midcondyle anteroposterior length
19. Intercondylar fossa depth	The horizontal distance between the most anterior point of the inferior border of the intercondylar notch and the tangent to the posterior surface of the femoral condyles
20. Chord	In a medial view, the distance between the deepest (most dorsal) point of the anterior femoral contour (just distal to the greater trochanter) and the maximum concavity on the anterior distal shaft (just proximal to the patellar surface)

Measurement	Description
21. Subtense (Martin #27)	The perpendicular distance from the chord line to the anterior shaft at maximum curvature
22. Point of maximum curvature	The position of the point of the femur's maximum curvature
[23. Total cross-sectional area (mm^2)]	The total cross-sectional area of the midshaft
[24. Cortical cross-sectional area (mm^2)]	The cortical area of the midshaft's cross section
[25. Cortical area ratio]	(Cortical area/total area) × 100

*All citations of Martin in this table refer to Martin (68).

†Trinkaus (32)

‡De Groote (36)

SI 4 Table 2: Description of tibia measurements. The numbering of the measurements corresponds to the numbers in SI 4 Fig. 2; note that the measurements in brackets are not depicted in the figure. Unless otherwise specified, measurements are in millimeters

Measurement	Description
[1. Actual length]	The distance between the most proximal point and the most distal point of the tibia
2. Tibia biomechanical length	The distance between the talar facet and the articular condyle facet
3. Maximum tibial length	For 'Ein Qashish, a composite consisting of the actual length of the left tibia and the medial malleolus length of the right tibia

Measurement	Description
4. Proximal epiphyseal breadth	The distance between the most lateral end and the most medial end of the proximal tibia
[5. AP diameter at ⅓ of total tibial length]	The anteroposterior diameter of the tibial shaft at one-third of its length, at the nutrient foramen
[6. Mediolateral diameter at ⅓ of total tibial length]	The mediolateral diameter of the tibial shaft at ⅓ of its length
[7. Cnemic index at ⅓ of total tibial length]	(Mediolateral diameter at ⅓ tibial length/anteroposterior diameter at ⅓ tibial length) × 100
[8. Midshaft AP diameter]	The anteroposterior diameter of the tibial shaft at 50% of its length
[9. Midshaft ML diameter]	The mediolateral diameter of the tibial shaft at 50% of its length
[10. Cnemic index at midshaft]	(Midshaft mediolateral diameter/midshaft anteroposterior diameter) × 100
[11. Midshaft circumference]	The circumference at the midshaft
[12. Robusticity index]	The square root of the product of the anteroposterior diameter and the mediolateral diameter at the midshaft, divided by the maximum tibial length, times 100
13. Distal tibia maximum ML breadth	The maximum mediolateral breadth of the distal tibia
14. Distal tibia maximum AP length	The maximum anteroposterior length of the distal tibia
15. Distal articular facet maximum ML breadth	The maximum mediolateral breadth of the distal articular facet

Measurement	Description
16. Distal articular facet, medial end, maximum AP length	The maximum anteroposterior length of the distal articular facet, at the medial end
17. Distal articular facet, lateral end, maximum AP length	The maximum anteroposterior length of the distal articular facet, at the lateral end
18. Medial malleolus length	The vertical distance between the distal end of the articular facet and the tip of the malleolus

SI 4 Table 3: Femur measurements of EQH-3, recent *H. sapiens*, *early H. sapiens*, and Neandertals.

Measurement	EQH-3	Recent *H. sapiens* $X \pm$ sd	Early *H. sapiens* $X \pm$ sd	Neandertals $X \pm$ sd
Femur bicondylar length (mm)	438	$443.4 \pm 26.3^{*}$ (Europeans); $426.5 \pm 34.2^{\dagger}$	$492 \pm 20.4^{*}$ (Skhul hominids); $460.5 \pm 36.7^{\ddagger}$ $456.1 \pm 34.2^{\dagger}$	$430.6 \pm 27.9^{*}$ $434.8 \pm 26.2^{\ddagger}$ $430.3 \pm 32.1^{\dagger}$
Femur biomechanical length (mm)	407	N/A	$454.3^{\S}$ (Skhul hominids)	$409.6^{\S}$
Neck shaft angle (degrees)	119	$128.5 \pm 4.7^{*}$ $127.4 \pm 5.7^{\dagger}$	$130 \pm 7.0^{*}$ $124.3 \pm 7.6^{\dagger}$	$120 \pm 5.3^{*}$ $118.7 \pm 5.2^{\dagger}$
Torsion angle (degrees)	19	$16.7 \pm 6.9^{\dagger}$	$11.2 \pm 9.9^{\dagger}$	$10.4 \pm 14.9^{\dagger}$

Measurement	EQH-3	Recent *H. sapiens*<br>*X* ± sd	Early *H. sapiens*<br>*X* ± sd	Neandertals<br>*X* ± sd
Femoral AP midshaft diameter (mm)	32.4	29.5 ± 2.8[*]	33.8 ± 3.7[*]	29.2 ± 3.9[*]
Femoral ML midshaft diameter (mm)	32.7	27.6 ± 2.2[*]	27.3 ± 2.0[*]	29.4 ± 2.1[*]
Midshaft shape ratio (pilastric index)	99	107–119[*] (range of means of various human populations);<br>114.2 ± 19.1[†]	120 ± 14.9[§] (Skhul hominids);<br>128.4 ± 20.9[†]<br>117 ± 10.4[¶]	99.3[*]<br>103.0 ± 14.5[†]<br>102 ± 9.3[¶]
Midshaft circumference (mm)	98.17	84.0 ± 4.4 (males)[‖]<br>74.8 ± 4.0 (females)[‖]	N/A	89–108 (Shanidar 4–6)[#]
Subpilastric index	85.6	88.1 ± 15.7[†]	102.1 ± 18.8[†]	87.6 ± 9.8[†]
Robusticity index[*]	7.4	6.13–6.39[*] (range of means of various human populations)	6.19[*]	6.92 ± 0.16[*]
Robusticity index[†]	14.9	12.4 ± 1.1[†]	13.4 ± 0.9[†]	13.7 ± 1.0[†]
Maximum ML condylar breadth (mm)	73.9	76.2 ± 2.3 (male)[‖]<br>69.8 ± 2.1 (female)[‖]<br>88.6 ± 4.2 (male)[**]<br>78.5 ± 3.0 (female)[**]	81.6 ± 6.7 (Qafzeh & Skhul hominids)[††]	84.3 ± 8.0[††]

Measurement	EQH-3	Recent *H. sapiens*<br>X ± sd	Early *H. sapiens*<br>X ± sd	Neandertals<br>X ± sd
ML breadth of lateral condyle (mm)	22.7	25.3 ± 2.6[‡‡]	N/A	N/A
AP length of lateral condyle (mm)	62.5	63.7 ± 5.1[‡‡]<br>61.1 ± 3.3 (male)[**]<br>55.4 ± 2.1 (female)[**]	68.8 ± 5.9 (Skhul hominids)[§§]	66.4 ± 6.4[¶¶] (*N* = 7)
ML breadth of medial condyle (mm)	31.0	26.7 ± 2.7[‡‡]	N/A	N/A
AP length of medial condyle (mm)	61.6	61.1 ± 3.4 (male)[**]<br>55.9 ± 2.9 (female)[**]	61.3 ± 7.2 (Skhul hominids)[§§]	63.3 ± 8.7[¶¶] (*N* = 7)
Epiphyseal breadth ratio	16.9	17.1 ± 1.3[†]	17.1 ± 1.2[†]	18.9 ± 1.4[†]
Intercondylar fossa breadth (mm)	10.7	22 ± 1.8 (male)[**]<br>18.7 ± 1.0 (female)[**]	N/A	N/A
Intercondylar fossa depth (mm)	24.8	27.8 ± 1.6 (male)[**]<br>23.7 ± 2.0 (female)[**]	N/A	N/A
Chord (mm)	357	254–343[##] (range of means of various human populations)	334.8 ± 31.0[##]	317.6 ± 28.3[##]
Subtense (mm)	26.6	6.2–11.7[##] (range of means of various human populations)	14.3 ± 3.0[##]	15.5 ± 3.4[##]
Point of maximum curvature (mm)	228	N/A	151.2 ± 21.1[##]	190.5 ± 37.0[##]

Measurement	EQH-3	Recent *H. sapiens*<br>*X* ± sd	Early *H. sapiens*<br>*X* ± sd	Neandertals<br>*X* ± sd
Total cross-sectional area (mm^2)	749	456 ± 62 (African-American females)<br>440 ± 58 (Caucasian females)[||||]	606.8 ± 95.4[***]	661.0 ± 54.3[***]
Cortical cross-sectional area (mm^2)	595	N/A	459.5 ± 89.3[***]	523.5 ± 53.6[***]
Cortical area percentage	79.4	N/A	75.6 ± 7.3[***]	79.1 ± 2.7[***]

[*]Trinkaus (32)

[†]De Groote (36)

[‡]Walker, et al. (80)

[§]Trinkaus & Ruff (39)

[¶]Garralda, et al. (97)

[#]Trinkaus (33)

[||]Gaikwad & Nikam (98)

[**]Terzidis, et al. (99)

[††]Adapted from Vandermeersch (79)

[‡‡]Ho, et al. (100)

[§§]Adapted from McCown & Keith (21)

[¶¶]Adapted from Heim (34)

[##]Shackelford & Trinkaus (35)

[||||]Nelson, et al. (101)

[***]Beauval, et al. (81)

SI 4 Table 4. Tibia measurements of EQH-3, recent *H. sapiens*, early *H. sapiens*, and Neandertals.

Measurement	EQH-3		Recent *H. sapiens*	Early *H. sapiens*	Neandertals
	Left tibia	Right tibia			
Actual length (mm)	338.5	290.7	N/A	N/A	N/A
Tibia biomechanical length (mm)	332	N/A	373[*]	409 (Skhul 4); 362 (Cro-Magnon)[*]	305 (Spy); 298 (Tabun 1)[*]
Maximum tibial length (mm)	338.5 +18.5 = 357.0	N/A	(349–376) ± 26[†]	387 ± 29[‡] 391 ± 27[†]	340 ± 26[‡] 337 ± 23[†]
Proximal epiphyseal breadth (mm)	77	N/A	70.6 ± 5.5 [§]	N/A	80 (Shanidar 2)
AP diameter at ⅓ of total tibial length (mm)	38	N/A	33.6 ± 4.0 [§]	N/A	38.4[‡]
ML diameter at ⅓ of total tibial length (mm)	23	N/A	22.6 ± 1.5[§]	N/A	26.6[‡]

**Measurement**	**EQH-3**		**Recent *H. sapiens***	**Early *H. sapiens***	**Neandertals**
	**Left tibia**	**Right tibia**			
Cnemic index at ⅓ of total tibial length	60	N/A	64–72 ± 6‡ (range of means of various human populations)	64 ± 6‡	69 ± 6‡
Midshaft AP diameter (mm)	36.9	35.32	N/A	35 ± 2.1¶ (Skhul & Qafzeh hominids)	33.1 ± 3.5#
Midshaft ML diameter (mm)	21.5	21.21	N/A	24.8 ± 1.5¶ (Skhul &Qafzeh hominids)	23.1 ± 2.3#
Cnemic index at midshaft	58	60	59.6–74.8# (range of means of various human populations)	78.8 ± 2.3 (Skhul & Qafzeh hominids)#	68.9 ± 6.6 (males)#<br>74.8 ± 0.5 (females)#<br>69 ± 6‡ (pooled sample)

**Measurement**	**EQH-3**		**Recent *H. sapiens***	**Early *H. sapiens***	**Neandertals**
	**Left tibia**	**Right tibia**			
Midshaft circumference (mm)	103	96	N/A	84–96 ± 10[¶]	86 ± 8[¶]
Robusticity index	8.0	7.7	6.2–7.1 ± 0.5[‡] (range of means of various human populations)	6.7 ± 0.4[‡]	8.1 ± 0.5[‡]
Distal tibia maximum ML breadth (mm)	N/A	51.14	N/A	N/A	54 (Shanidar)[‖]
Distal tibia maximum AP length (mm)	N/A	36.9	N/A	N/A	39.3 (Shanidar)[‖]
Distal articular facet maximum ML breadth (mm)	N/A	29.53	N/A	N/A	31.3–32 (Shanidar)[‖]

Measurement	EQH-3		Recent *H. sapiens*	Early *H. sapiens*	Neandertals
	**Left tibia**	**Right tibia**			
Distal articular facet, medial end, maximum AP length (mm)	N/A	25.73	N/A	N/A	N/A
Distal articular facet, lateral end, maximum AP length (mm)	N/A	33.5	N/A	N/A	28.6–33.0 (Shanidar)[‖]
Medial malleolus length (mm)	N/A	18.5	N/A	N/A	N/A
Crural index: (tibial length/femoral length) × 100		81.5	84–90 ± 4.3[**] (range of means of various human populations)	85.1 ± 2.8[**] (*N* = 25)	78.8 ± 1.7[**] (*N* = 9)

[*]McCown & Keith (21)

[†]Walker, et al. (80)

[‡]Lovejoy & Trinkaus (37)

[§]González-Reimers, et al. (102)

[¶]Stringer, et al. (38)

[#]Trinkaus (69)

[||]Trinkaus (33)

[**]Porter (103)

SI 4 Table 5. The femur and tibiae of Neandertal males and females compared to the EQH-3 femur and tibiae.

**Measurement**	**Male** $X \pm$ sd (N)	**Female** $X \pm$ sd (N)	**EQH-3**
Femoral bicondylar length	442.8 ± 20.4 (9)[*]	400.3 ± 14.3 (3)[*]	438
Tibial length	354.4 ± 19.3 (8)[*]	310.0 ± 9.5 (3)[*]	357
Femur robusticity index	7.19 ± 0.59 (8)[*]	6.91 ± 0.62 (3)[*]	7.4
Tibial robusticity index	8.24 ± 0.4 (7)[*]	7.78 ± 0.49 (3)[*]	7.7–8.0
Tibial cnemic index	68.9 ± 6.6[†]	74.8 ± 0.5[†]	61

[*]Trinkaus (69)

[†]Trinkaus (33)

SI 4 Table 6. Estimated stature of EQH-3.

**Estimation Method**	**Reference**	**Population**	**Formula**	**Stature estimation for EQH-3**
Femur length (FEL)	Sjøvold (104)	All races	2.71 × FEL + 45.86	164.6 (± 4.49)
Femur length	Trotter & Gleser (89)	European-American males	2.38 × FEL + 61.41	165.6 (± 3.27)

Estimation Method	Reference	Population	Formula	Stature estimation for EQH-3
Femur length	Trotter & Gleser (89)	African-American males	2.11 × FEL + 70.35	162.8 (± 3.94)
Tibia length (TL)	Sjøvold (104)	All races	3.29 × TL + 47.34	165 (± 4.15)
Tibia length	Trotter & Gleser (89)	European-American males	2.52 × TL + 78.62	168.6 (± 3.37)
Tibia length	Trotter & Gleser (89)	African-American males	2.19 × TL + 86.02	164.2 (± 3.78)
Tibia length	Auerbach & Ruff (91)	North American Arctic males	2.55 × TL + 69.51	160.5 (± 2.99)
(Femur and tibia) length	Trotter & Gleser (89)	European-American males	1.39 × (FEL + TL) + 53.2	163.7 (± 3.55)
(Femur and tibia) length	Trotter & Gleser (89)	African-American males	1.26 × (FEL + TL) + 59.72	159.9 (± 3.28)
Femur length and tibia length	Auerbach & Ruff (91)	North American Arctic males	(1.28 × FEL) + (1.26 × TL) + 59.86	160.9 (± 2.62)
Femur length and tibia length	Auerbach & Ruff (91)	North American Great Plains males	(1.88 × FEL) + (0.76 × TL) + 54.13	163.6 (± 1.94)

Estimation Method	Reference	Population	Formula	Stature estimation for EQH-3
Mean of stature values for EQH-3				163.6

SI 4 Table 7. Comparison of stature estimation for EQH-3, recent *H. sapiens*, early *H. sapiens*, Neandertals, and Sima de los Huesos hominins.

Population	EQH-3	Recent *H. sapiens*[*]	Early *H. sapiens*[*]	Neandertals[*]	Sima de los Huesos hominins[*]
Males & females	163.6	N/A	177.45	160.6	163.6
Males	N/A	Range of means for 124 human populations: 144.1–184.9	185.1 ± 7.1	166.7 ± 5.9	169.5 ± 4.0
Females	N/A	Range of means for 124 human populations: 137.0–167.7	169.8 ± 6.5	154.5 ± 4.6	157.7 ± 2.0

[*]Data from Carretero, et al. (40)

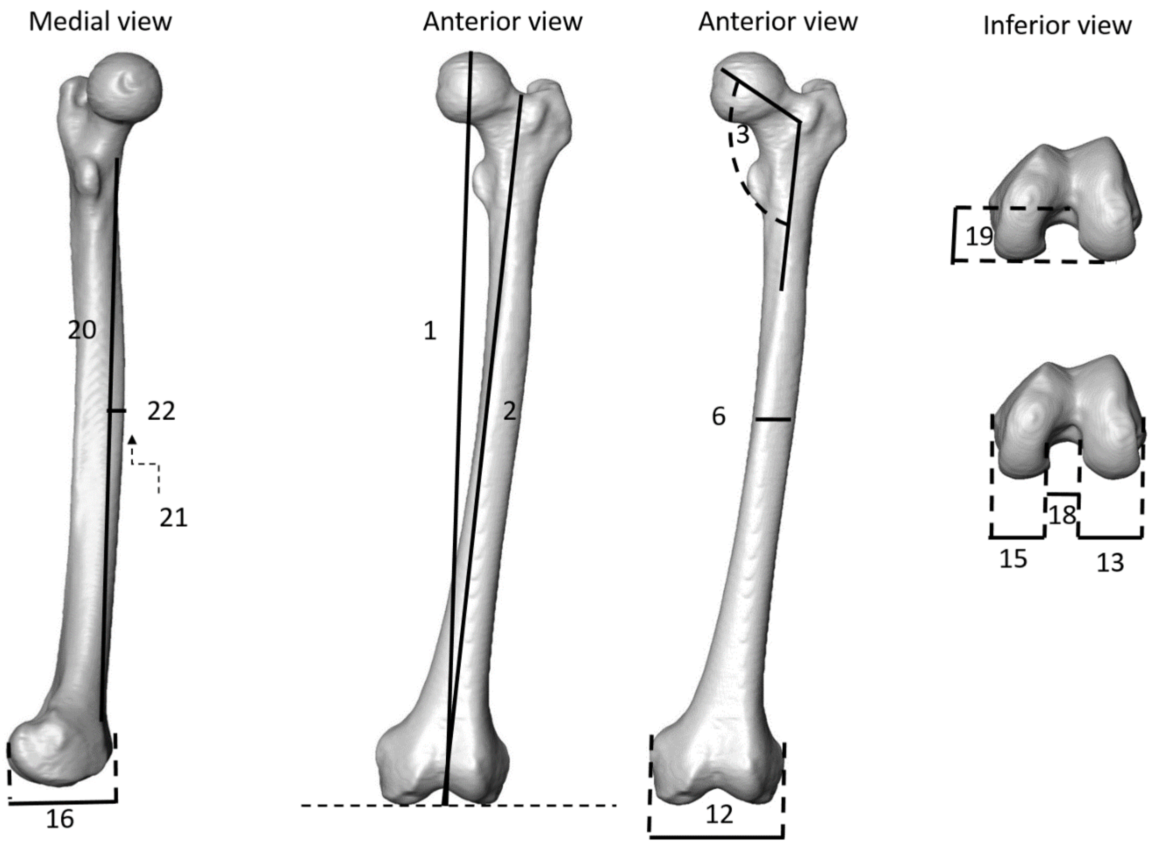

SI 4 Fig. 1. Femoral measurements. The numbers correspond to the measurement numbers in SI 4 Table 1.

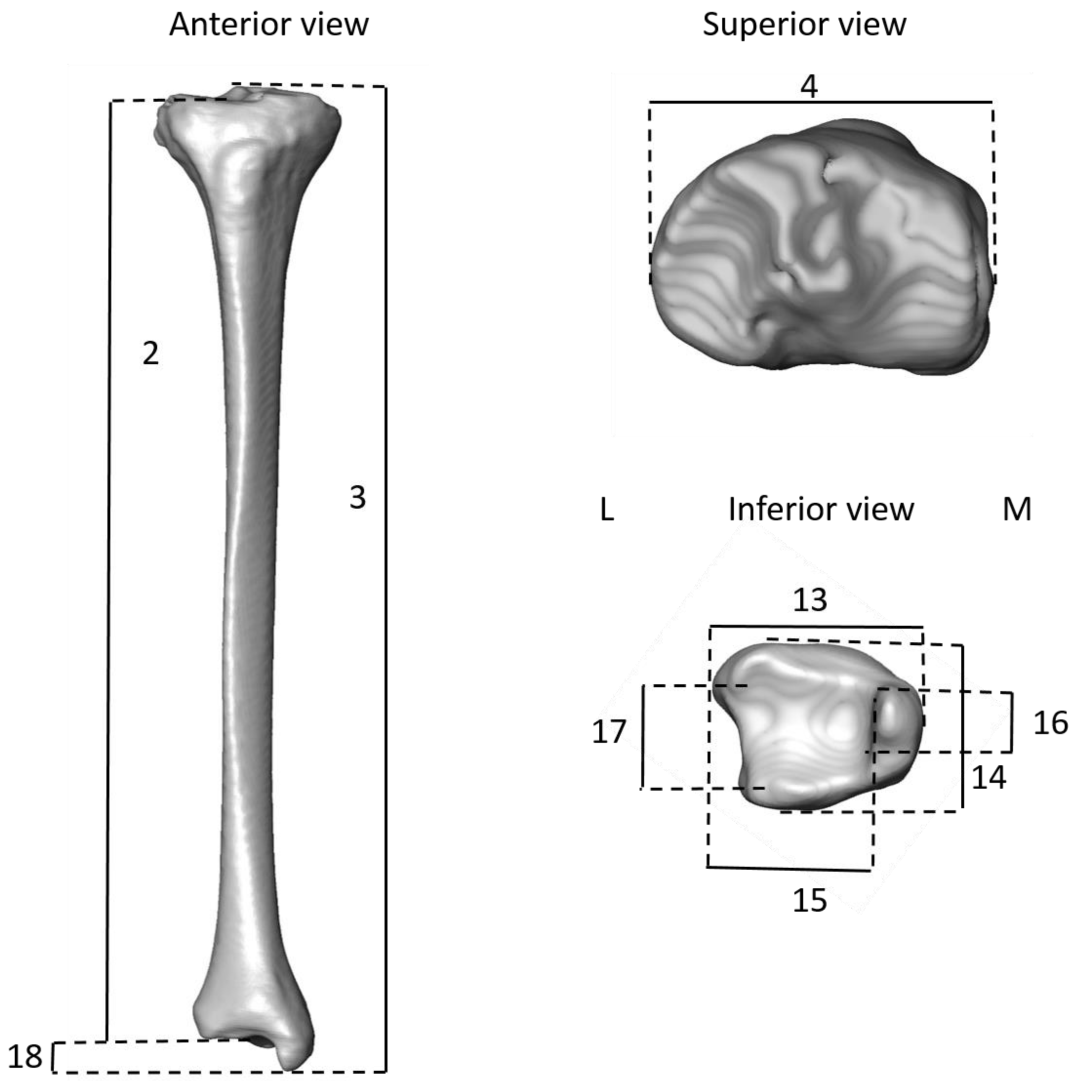

SI 4 Fig. 2. Tibial measurements. The numbers correspond to the measurement numbers in SI 4 Table 2.

References

70. Nir. N. Bones and formation processes in the open-air Middle Paleolithic site of Ein Qashish. *MA thesis, Institute of Archaeology*. (The Hebrew University of Jerusalem, 2016).
71. Davidovich. U., Porat, N., Gadot, Y., Avni, Y., & Lipschits, O. Archaeological investigations and OSL dating of terraces at Ramat Rahel, Israel. *J. Field. Archaeol*. **37**, 192–208 (2012).
72. Murray, A.S., & Wintle, A.G. Luminescence dating of quartz using an improved single aliquot regenerative-dose protocol. *Radiat. Meas*. **32**, 57–73 (2000).
73. Galbraith, R.F., & Roberts, R.G. Statistical aspects of equivalent dose and error calculation and display in OSL dating: an overview and some recommendations. *Quat. Geochronol*. **11**, 1–27 (2012).

74. Nambi, K.S.V., & Aitken, M.J. Annual dose conversion factors for TL and ESR dating. *Archaeometry* **28**, 202–205 (1986).
75. Faershtein, G., Porat, N., Avni, Y., & Matmon, A. Aggradation-incision transition in arid environments at the end of the Pleistocene: an example from the Negev Highlands, southern Israel. *Geomorphology* **253**, 289–304 (2016).
76. Frayer, D.W. *Evolution of the Dentition in Upper Paleolithic and Mesolithic Europe.* University of Kansas Publications in Anthropology (vol. 10), (Lawrence, Kansas, 1978).
77. Suzuki, H., & Takai, F., Eds. *The Amud Man and His Cave site* (Academic Press of Japan, 1970).
78. Wolpoff, M.H. The Krapina dental remains. *Am. J. Phys. Anthropol.* **50**, 67-113 (1979).
79. Vandermeersch, B. *Les Hommes Fossiles de Qafzeh (Israël)* (Centre National de la Recherche Scientifique, Paris,1981).
80. Walker, M.J., Ortega, J., Parmová, K., López, M.V., & Trinkaus, E. Morphology, body proportions, and postcranial hypertrophy of a female Neandertal from the Sima de las Palomas, southeastern Spain. *Proc. Natl. Acad. Sci.* **108**, 10087–10091(2011).
81. Beauval, C., et al. A late Neandertal femur from les Rochers-de-Villeneuve, France. *Proc. Natl. Acad. Sci. USA* **102**, 7085–7090 (2005).
82. Stringer, C., & Gamble, C. *In Search of the Neanderthals: Solving the Puzzle of Human Origins* (Thames and Hudson,1993).
83. Ruff, C.B. Climatic adaptation and hominid evolution: the thermoregulatory imperative. *Evol. Anthropol*. 2, 53–60 (1993).
84. Weaver, T.D. The shape of the Neandertal femur is primarily the consequence of a hyperpolar body form. *Proc. Natl. Acad. Sci*. **100**, 6926–6929 (2003).
85. Trinkaus, E., & Ruff, C.B. Diaphyseal cross-sectional geometry of Near Eastern Middle Palaeolithic humans: The Tibia. *J. Archaeol. Sci*. **26**, 1289–1300 (1999).
86. Patte, E. *Les Néanderthaliens*. (Paris, Masson et Cie, 1955).
87. Taylor, J.V. *The Neanderthal Tibia*. PhD thesis. Columbia University (Ann Arbor, University Microfilms, 1968).
88. Trotter, M. Estimation of stature from intact long limb bones. *Personal Identification in Mass Disasters.* (ed. T.D. Stewart) 71-84 (Smithsonian Institution, National Museum of Natural History, Washington, DC, 1970).
89. Trotter, M., & Gleser, G.C. Estimation of stature from long bones of American Whites and Negroes. *Am. J. Phys. Anthropol*. **10**, 463–514 (1952).
90. Feldesman, M.R., & Lundy, J.K. Stature estimates for some African Plio-Pleistocene fossil hominids. *J. Hum. Evol*. **17**, 583–596 (1988).
91. Auerbach, B.M., & Ruff, C.B. Stature estimation formulae for indigenous North American populations. *Am. J. Phys. Anthropol.* **141**, 190–207 (2010).
92. Lieberman, D.E., Devlin, M.J., & Pearson, O.M. Articular area responses to mechanical loading: effects of exercise, age, and skeletal location. *Am. J. Phys. Anthropol.* **116**, 266–277 (2001).
93. Stäubli, H.U., & Rauschning, W. Tibial attachment area of the anterior cruciate ligament in the extended knee position. *Knee Surg. Sport. Tr. A.* **2**, 138–146 (1994).
94. Perugia, D., Basiglini, L., Vadala, A., & Ferretti, A. Clinical and radiological results of arthroscopically treated tibial spine fractures in childhood. *Int. Orthop.* **33**, 243–248 (2009).

95. Furlan, D., Pogorelić, Z., Biočić, M., Jurić, I., & Meštrović, J. Pediatric tibial eminence fractures: arthroscopic treatment using K-wire. *Scand. J. Surgery* **99**, 38–44 (2010).
96. Anderson, A.F., & Anderson, C.N. Correlation of meniscal and articular cartilage injuries in children and adolescents with timing of anterior cruciate ligament reconstruction. *Am. J. Sports Med*. **43**, 275–281 (2015).
97. Garralda, M.D., Galván, B., Hernández, C.M., Mallol, C., Gómez, J.A., & Maureille, B. Neanderthals from El Salt (Alcoy, Spain) in the context of the latest Middle Palaeolithic populations from the southeast of the Iberian Peninsula. *J. Hum. Evol*. **75**, 1–15 (2014).
98. Gaikwad, K.R., & Nikam, V.R. Sexual Dimorphism in Femur. IOSR *J. Dental. Medic. Sci*. **13**, 4–9 (2014).
99. Terzidis, I., et al. Gender and side-to-side differences of femoral condyles morphology: osteometric data from 360 Caucasian dried femori. *Anat. Res. Int*. doi:10.1155/2012/679658 (2012).
100. Ho, W.P., Cheng, C.K., & Liau, J.J. Morphometrical measurements of resected surface of femurs in Chinese knees: correlation to the sizing of current femoral implants. *The Knee* **13**, 12–14 (2006).
101. Nelson, D.A., Barondess, D.A., Hendrix, S.L., & Beck, T.J. Cross-Sectional Geometry, Bone Strength, and Bone Mass in the Proximal Femur in Black and White Postmenopausal Women. *J. Bone. Miner. Res*. **15**, 1992–1997 (2000).
102. González-Reimers, E., et al. Bending and torsional strengths of the tibia vs. simple anthropometric variables among the prehispanic population of El Hierro (Canary Islands). *Eur. J. Anat*. **18**, 8–15 (2014).
103. Porter, A.M.W. Modern human, early modern human and Neanderthal limb proportions. *Int. J. Osteoarchaeol.* **9**, 54–67 (1999).
104. Sjøvold, T. Estimation of stature from long bones utilizing the line of organic correlation. *Hum. Evol*. **5**, 431–447 (1990).
